# Essential gene acquisition destabilizes plasmid inheritance

**DOI:** 10.1371/journal.pgen.1009656

**Published:** 2021-07-12

**Authors:** Tanita Wein, Yiqing Wang, Myriam Barz, Fenna T. Stücker, Katrin Hammerschmidt, Tal Dagan

**Affiliations:** Institute of General Microbiology, Kiel University, Kiel, Germany; University of Oxford, UNITED KINGDOM

## Abstract

Extra-chromosomal genetic elements are important drivers of evolutionary transformations and ecological adaptations in prokaryotes with their evolutionary success often depending on their ‘utility’ to the host. Examples are plasmids encoding antibiotic resistance genes, which are known to proliferate in the presence of antibiotics. Plasmids carrying an essential host function are recognized as permanent residents in their host. Essential plasmids have been reported in several taxa where they often encode essential metabolic functions; nonetheless, their evolution remains poorly understood. Here we show that essential genes are rarely encoded on plasmids; evolving essential plasmids in *Escherichia coli* we further find that acquisition of an essential chromosomal gene by a plasmid can lead to plasmid extinction. A comparative genomics analysis of *Escherichia* isolates reveals few plasmid-encoded essential genes, yet these are often integrated into plasmid-related functions; an example is the GroEL/GroES chaperonin. Experimental evolution of a chaperonin-encoding plasmid shows that the acquisition of an essential gene reduces plasmid fitness regardless of the stability of plasmid inheritance. Our results suggest that essential plasmid emergence leads to a dose effect caused by gene redundancy. The detrimental effect of essential gene acquisition on plasmid inheritance constitutes a barrier for plasmid-mediated lateral gene transfer and supplies a mechanistic understanding for the rarity of essential genes in extra-chromosomal genetic elements.

## Author summary

Mobile genetic elements have been extensively studied due to their role as agents of genetic innovation and rapid adaptation in prokaryotes. Specifically, prokaryotic plasmids have been the focus of investigation in the context of bacterial survival under growth limiting conditions with the prime example of resistance to antibiotics and heavy metals. In contrast, plasmids that encode for functions that are essential to their host viability are rarely described. We investigate the evolution of plasmids that encode for genes previously identified as essential for bacterial life. Our analysis of *Escherichia* isolates reveals only few plasmid-encoded essential genes, which likely function in the plasmid rather than the host life cycle. Following the evolution of plasmids encoding an essential gene in *Escherichia coli* in real time, we further find that the acquisition of a chromosomal essential gene may lead to plasmid loss. Our study supplies data and a mechanistic understanding on the rarity of essential genes in mobile genetic elements. We conclude that prokaryotic plasmids are rarely essential for their bacterial host.

## Introduction

Plasmids are autonomously replicating genetic elements that are prominent in prokaryotes and have been studied extensively due to their contribution to lateral gene transfer. Plasmid invasion is often accompanied by the acquisition of novel traits that enable bacteria to survive under specific conditions or colonize specific ecological niches. Plasmids carrying beneficial genes, e.g., those that supply resistance against antibiotics (e.g., [1,2]) or heavy metals (e.g., [3,4]) enable their host to survive under transient selective conditions. A strong selection for the plasmid-encoded trait over many generations was shown to lead to co-adaptation of the plasmid and the host, and the evolution of stable plasmid inheritance [5–9]. Nonetheless, environmental conditions are rarely constant; a decrease in the strength of selection for plasmid-encoded beneficial genes–e.g., due to fluctuating abundance of growth limiting factors–may lead to plasmid loss and extinction. In contrast, plasmids that supply the host with essential functions, i.e., whose benefit to the host is less dependent on temporary environmental conditions, may persist over longer time scales and become an integral component of the lineage genome in the form of chromids [10]. The level of gene essentiality is defined as the extent to which a gene is required for the reproduction of an organism [11]. Borrowing that definition, plasmids that encode for essential genes may be thus considered as vital for the host proliferation. Examples include plasmids encoding for genes along the biosynthesis pathways of essential amino acids (e.g. [12,13]) and components of the ribosome machinery [14]. Nevertheless, it has been previously suggested that plasmids encoding essential genes should be rare in nature as their adaptation to the host is accompanied by reduction of host fitness due to plasmid loss [15]. Thus, the evolution of plasmids encoding essential genes may depend on the evolution of stable plasmid-host interactions.

In the absence of selection for plasmid-encoded genes, plasmid persistence largely depends on plasmid stability that comprises plasmid replication and segregation. Indeed, plasmids that have a negligible (i.e., neutral) effect on host fitness may evolve stable inheritance and consequently gain long-term persistence in the population–also in the absence of positive selection [16]. Stably inherited plasmids may serve as precursors for the evolution of larger plasmids carrying beneficial or essential genes. Notably, the essential plasmids reported in the literature so far are characterized by a stable vertical inheritance. The evolution of such essential plasmids may thus follow two alternative routes [17]: *essentiality first*, where the plasmid is initially essential to the host; a plasmid carrying an essential function may persist in the population over long timescales and eventually evolve a stable plasmid reproduction within the host. In the second route, termed *stability first*, a plasmid that initially evolved a stable reproduction cycle may subsequently evolve into an essential plasmid following an essential gene gain. Notwithstanding, as essential genes are typically encoded in the chromosomes [18], the presence of an essential gene on a plasmid may be comparable to the effect of gene duplication. In addition, the expression level of plasmid-encoded genes may be amplified following an increase in plasmid copy number (e.g., [19]). Therefore, the evolution of essential genes on plasmids is likely under selection pressure that is typical to gene duplication, such as dose effect (reviewed in [20]). Evidently, most genes (89 ± 8%) in prokaryotic genomes are found in a single copy [21].

Here we combine computational genomic analysis and experimental work to investigate the evolution of essential plasmids in the genus *Escherichia*. First, we investigate the frequency of shared genes between plasmids and chromosomes in *Escherichia* as well as the distribution of essential genes in *Escherichia* plasmids. Furthermore, we compare both the *essentiality* and *stability first* evolutionary scenarios using an evolution experiment in *E*. *coli* where we follow the evolution of stable and unstable plasmids encoding the chaperonin that is indispensable in *E*. *coli*.

## Results

### Plasmid gene content is rarely shared with the host chromosome

**Fig 1:**
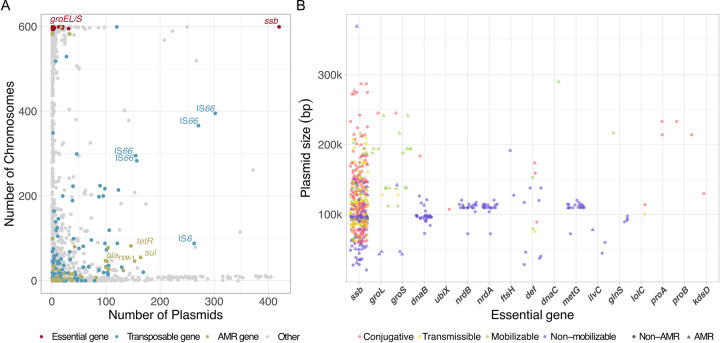
The distribution of plasmid-encoded essential genes on plasmids and chromosomes. **A**, The distribution of *Escherichia* protein families on plasmids in chromosomes (in grey). Essential genes (red) are found in most of the chromosomes and rarely on plasmids, with *ssb* as an exception. Transposable elements (blue) are frequent on both chromosomes and plasmids. IS*66* is the most widely spread gene in *Escherichia* strains. IS*66* was split into several protein families in our analysis, which are depicted by multiple data points. Antibiotic resistance (AMR) genes (yellow) are presented for comparison. The AMR genes can be roughly divided into two groups: the first group aligns along the y-axis hence it is more frequently found on chromosomes; those chromosome-encoded AMR genes are typically related to persistence and resilience functions. The second group aligns along the x-axis hence it is more frequently found on plasmids; those plasmid-encoded genes are typically related to antibiotics resistance functions (Wein et al. 2020). **B**, Distribution of plasmid size, mobility group and AMR for plasmids encoding an essential gene. Most of the *ssb*-coding plasmids are conjugative (red) while the majority of *groEL/S* coding plasmids are mobilizable (green). The remaining essential genes are encoded on plasmids that are often non-mobilizable (and non-AMR) (purple).

To study the distribution of chromosomal homologs to plasmid genes we examined isolates of *Escherichia* that are available as complete genome sequences at NCBI (S1 Table). Of the total 599 isolates, 416 isolates harbor between 1 and 10 plasmids. All 88,493 protein-coding genes encoded on plasmids were clustered into 4,780 proteins families by sequence similarity. Notably, we did not detect any universal plasmid gene–the largest plasmid protein family includes homologs from 41% (425/1035) plasmids. Additionally, we identified chromosomal homologs to the plasmid protein families using a sequence similarity search. This revealed that 2,682 (56%) of the plasmid protein families had no chromosomal homolog, while the remaining 2,098 (44%) families had a chromosomal homolog in at least one *E*. *coli* isolate. Previously it has been suggested that gene duplication via gene transfer may be disadvantageous in prokaryotes due to dose effect (e.g., [22,23]). Indeed, the distribution of protein family members on chromosomes and plasmids shows that the level of gene sharing between both replicon types is rather low (Fig 1A). A total of 383 (8%) protein families have a chromosomal homolog in a single isolate, and 544 (11%) of the plasmid protein families have a chromosomal homolog in 2–320 isolates (S1 Fig). Thus, in total 927 (19%) of the plasmid protein families have a chromosomal homolog in the same isolate (i.e., the plasmid host), of those, 133 (3%) families correspond to transposable elements (Fig 1A). The most abundant protein family among the transposable elements is that of IS*66* that is found in varying copy numbers on 395 examined chromosomes and 302 plasmids.

### Essential genes are rarely encoded on plasmids

To study the evolution of essential genes encoded on plasmids, we examined the distribution of genes previously recognized as essential in *E*. *coli* on chromosomes and plasmids, where the extent of gene essentiality may depend on the environmental and nutritional conditions. The set of essential genes we tested here comprises 504 protein-coding genes (S2 Table); of those, 394 protein-coding genes were identified as essential in *E*. *coli* K-12 under standard laboratory conditions via gene knockout [24] or transposon mutagenesis [25]. Additional 110 protein-coding genes were identified as essential in a collection of 18 *E*. *coli* isolates by CRISPR interference of gene expression ([18]; only genes identified as essential in at least one out of the three tested growth media were included; S2 Table). Searching for plasmid-encoded homologs to the essential genes using sequence similarity revealed that only 17 (3%) of the essential genes had a homolog on plasmids (Fig 1A and Table 1). The 464 *Escherichia* plasmids encoding essential genes are typically of medium plasmid size (median: 106Kb), 32% of them are non-mobile and only 4% carry in addition an antibiotic resistance gene (Fig 1B). Importantly, all isolates where we identified a plasmid homolog of an essential gene also harbored the chromosomal copy of that gene (except one isolate carrying only *metG* on the plasmid).

**Table 1:** Data and phylogenetic analysis of essential genes encoded on plasmids. Essential list indicates the source of essential genes: TKP is TraDIS-Keio-PEC, TP is TraDIS-PEC, T is TraDIS (Goodall et al. 2018); LB, M9 and GMM means this gene is essential in each of the three medias (Rousset et al. 2020). Relaxed selection shows evidence for relaxed selection on plasmid homologs’ branch was found using HyPhy-RELAX (Murrell et al. 2012). Tree topology shows the conclusions from the phylogenetic reconstruction of essential genes on plasmids and chromosomes: *split between plasmids and chromosomes* describe phylogenies that had a deep split between plasmid and chromosomal homologs (i.e., they are diverged); LGT events from chromosome to plasmid are labeled by *transfer to plasmid*; LGT events from plasmid to chromosome are labeled by *transfer to chromosome*; *mixed* is used to label phylogenies that contain deep divergence and LGT events. For gene trees with evidence for gene transfer (*groEL*, *groES*), we further tested the support in the plasmid/chromosome split while excluding the putatively transferred gene. In all tested cases the result showed that the constrained tree could not be rejected in a topology test, thus validating the gene transfer inference. All of the transfer event from plasmid to chromosome are better described as translocation of the plasmid homolog to chromosome.

Gene	Product	Essential list	No. Plasmids	No. Isolates	Relaxed selection	Tree topology
*ssb*	single-stranded DNA-binding protein	TKP	420	320	n.a.	Mixed
*groL*	chaperonin GroEL	TP	11	11	On plasmid branch	One translocation to Chromosome
*groS*	co-chaperone GroES	TKP	13	13	No evidence	One translocation to Chromosome
*dnaB*	replicative DNA helicase	TKP	31	31	No evidence	Two translocations to Chromosome
*ubiX*	aromatic acid decarboxylase	T	1	1	n.a.	Split between plasmids and chromosomes
*nrdB*	ribonucleotide-diphosphate reductase subunit beta	TKP	20	20	No evidence	Split between plasmids and chromosomes
*nrdA*	ribonucleoside-diphosphate reductase subunit alpha	TKP	19	19	On plasmid branch	Split between plasmids and chromosomes
*ftsH*	ATP-dependent metalloprotease FtsH	TKP	2	2	n.a.	Three transfers to plasmid
*def*	peptide deformylase	TKP	13	13	No evidence	Split between plasmids and chromosomes
*dnaC*	DNA replication protein DnaC	TKP	1	1	n.a.	Single transfer to plasmid
*metG*	methionine—tRNA ligase	TKP	19	19	No evidence	Mixed
*ilvC*	ketol-acid reductoisomerase, NAD(P)-binding	M9	3	3	No evidence	Split between plasmids and chromosomes
*glnS*	glutamine—tRNA ligase	TKP	5	5	n.a.	Five transfers to plasmid
*lolC*	lipoprotein-releasing ABC transporter permease subunit	TKP	2	2	n.a.	Split between plasmids and chromosomes
*proB*	gamma-glutamate kinase	M9	2	2	n.a.	Two transfers to plasmid
*proA*	gamma-glutamylphosphate reductase	M9	2	2	n.a.	Two transfers to plasmid
*kdsD*	D-arabinose 5-phosphate isomerase	LB, GMM	1	1	n.a.	Split between plasmids and chromosomes

**Fig 2:**
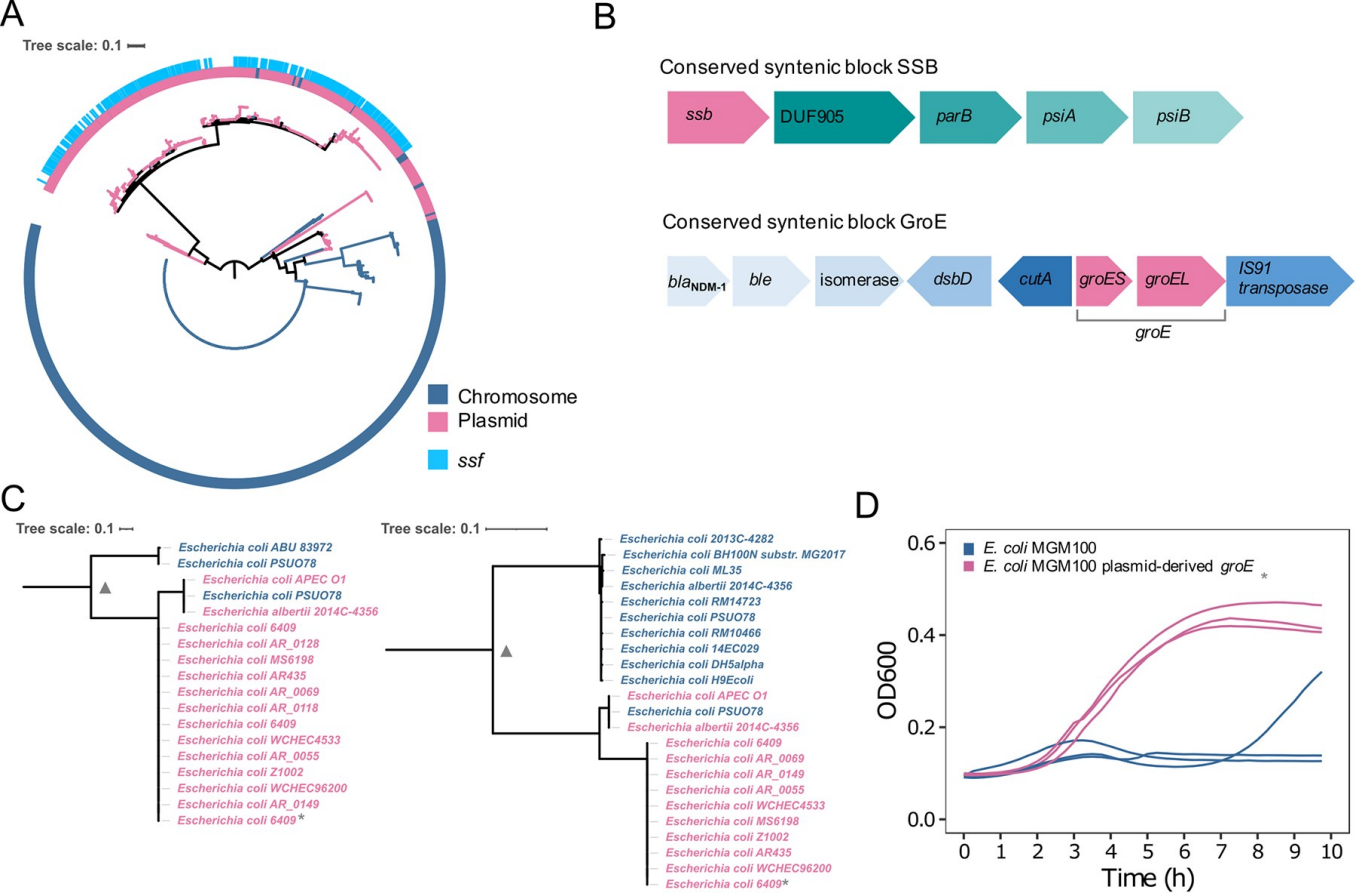
Phylogeny of plasmid-encoded essential genes in *Escherichia*. **A**, Phylogeny of the single-stranded DNA-binding protein Ssb. Ssb homologs encoded on plasmid are shown in pink and encoded on chromosome are shown in blue. The plasmid *ssb* gene termed *ssf* is most abundant across the plasmid Ssb homologs. **B**, The conserved neighborhood (conserved syntenic block, CSB) of *ssb* and the chaperone *groE* encoded on plasmids (S3 and S4 Tables). **C**, Phylogeny of the chaperonin GroES (left) and GroEL (right). The triangle symbol marks the branch split that was constrained in the test for an alternative tree topology. **D**, Growth measurements of *E*. *coli* MGM100 and MGM100 carrying the plasmid-derived GroE (marked by *). The exceptional growth behavior observed in one *E*. *coli* MGM100 replicate should be considered an outlier since we found no evidence for other explanations to that result.

The most frequent essential gene on plasmids is a single-strand DNA binding protein (*ssb*), which is nearly ubiquitous in *Escherichia* plasmids (Table 1 and Fig 2A). Ssb functions in DNA replication and recombination and is important for the establishment of conjugative plasmids in the recipient host cell [26]. The chromosomal *ssb* gene function can be complemented by a plasmid borne *ssb* (termed *ssf* [27]) in *E*. *coli* when present on a high copy-number plasmid (Porter and Black 1991). To investigate whether the high abundance of *ssb* on plasmids is the result of frequent lateral transfer, we examined the genomic neighborhood of *ssb* on plasmids. This revealed that the *ssb* genomic neighborhood is conserved in 64% (270/420) of the plasmids, comprising five genes that include a *parB* gene encoding type II partitioning system and two genes annotated as inhibitors of SOS response to conjugation (*psiA* and *psiB*; Fig 2B and S3 Table). Indeed, many of the plasmids encoding *ssb* are conjugative (43%; Fig 1B). A phylogenetic reconstruction of the *Escherichia* Ssb evolutionary history reveals a deep divergence between the chromosomal and plasmid homologs (Fig 2A). Additionally, the phylogenetic tree suggests several events of lateral gene transfer (LGT) of *ssb* from plasmids to chromosomes as indicated by eight chromosomal *ssb* homologs that branch with plasmid homologs (Fig 2A). To further validate the LGT events, we compared the reconstructed tree likelihood to an alternative constrained topology having a split between chromosomal and plasmid *ssb* homologs. Since the alternative *ssb* topology was rejected (P = 0.0074, using AU test), we conclude that, indeed, the *ssb* evolution included multiple LGT events. According to the inferred *ssb* phylogeny, a recent translocation of the plasmid-encoded *ssb* to the chromosome occurred in the seven strains, all of them retained the original chromosomal copy and lacked a plasmid-encoded *ssb*; these seven isolates thus have two *ssb* copies encoded in their chromosome. These *ssb* LGT events correspond to the translocation of an essential plasmid gene to the chromosome.

Further phylogenetic reconstruction of the remaining plasmid homologs to essential genes revealed evidence for a recent transfer from the chromosome to the plasmid in six of the protein families including *ftsH*, *dnaC*, *metG*, *glnS*, *proA* and *proB* (Table 1 and S2–S7 Figs). The evolution of those gene families is evidence for rare duplication of essential genes onto a resident plasmid. In contrast, the phylogenetic trees of nine essential genes including *groEL*, *groES*, *ubiX*, *nrdB*, *nrdA*, *def*, *ilvC*, *lolC* and *kdsD* revealed a clear split between the chromosomal and plasmid homologs (Table 1 and Figs 2C and S8–S14). In two of these gene families—*groEL* and *groES—*we observed gene translocation from plasmid to chromosome (Table 1 and Fig 2C). The divergence between the plasmid and the chromosomal homologs in these gene families suggests that the plasmid homologs have undergone a sub- or neo-functionalization (i.e., similar to the plasmid Ssb).

Previous studies suggested that laterally transferred genes are often non-functionalized due to codon-usage incompatibility or the absence of regulatory elements [28]. To investigate whether plasmid-encoded homologs of essential genes are pseudogenes, we inferred the strength of selection pressure on the plasmid branch relative to chromosome branch in all relevant protein families. Whereas our results revealed the presence of relaxed selection on branches leading to plasmid homologs in *groEL* and *nrdA* (Table 1 and S15 and S16 Figs), we could not detect evidence for non-functionalization of those genes (e.g., frameshift or truncation). Our results thus suggest that the observed relaxation of selection is likely associated with sub- or neo-functionalization of the essential genes encoded on plasmids.

### The plasmid-encoded chaperonin complements the chromosomal function

To further examine the evolution of plasmids encoding essential genes, we studied the implications of essential gene gain on a plasmid by focusing on the chaperonin genes *groEL* and *groES*. The chaperonin is universally encoded in eubacterial chromosomes with only rare exceptions (e.g., *Mollicutes* [29]) and plasmids encoding the chaperonin genes have been only reported in *Rhizobium* [30]. The *groES* and *groEL* genes are typically encoded within the *groE* operon. The GroE chaperonin plays a major role in the bacterial protein-folding pathway and is constitutively expressed in *E*. *coli* and essential for growth under all conditions [31]. An examination of the *groE* genomic neighborhood in *Escherichia* plasmids revealed a conserved neighborhood that includes, in addition to *groEL and groES* a *bla*_NDM-1_, *ble* (bleomycin binding protein Ble-MBL), phosphoribosylanthranilate isomerase, twin-arginine translocation (TAT) pathway signal sequence domain protein and *cutA* (divalent-cation tolerance protein) (Fig 2B and S4 Table). Indeed, a similar integron comprising *groE* has been previously reported in *Escherichia* [32,33]. Our results thus indicate that *groE* is likely not a pseudogene. To further test the function of the plasmid-derived *groE*, we cloned the gene sequence into a small model plasmid. The plasmid was then introduced into *E*. *coli* MGM100 (Fig 2D), which encodes the *groE*-operon under an inducible P_BAD_ promoter, hence it is only viable in medium supplemented with arabinose (i.e., P_BAD_ induction) or when it is complemented with *groE* [34]. In competition with *E*. *coli* MG1655, *E*. *coli* MGM100 has no measurable fitness disadvantage (S18 Fig). To test whether the plasmid-derived *groE* can complement the silenced *groE* in *E*. *coli* MGM100, we quantified the growth of this strain with and without supplemented arabinose. In addition, we recorded the overnight plasmid loss, as a plasmid that is essential to its host cannot be lost. Our results show that the plasmid can indeed complement the chromosomal GroE function, hence we conclude that the plasmid-encoded *groE* functions as a chaperonin.

### Essential gene acquisition destabilizes plasmid inheritance

Our results so far show that essential genes are rarely found on plasmids with the plasmid-encoded GroE in *Escherichia* as a rare exception. Such plasmids that encode an essential gene may evolve via two possible routes–in the *stability first* a preliminary stably inherited plasmid gains an essential gene, while in the *essentiality first* a plasmid that encodes an essential gene gains by that a stable inheritance. To study the evolutionary consequences of the acquisition of an essential chromosomal gene by plasmids, we conducted an evolution experiment with *E*. *coli* K12 MG1655 carrying small plasmids encoding a chromosomal *groE* copy. To investigate the implications of plasmid stability versus essentiality in the evolution of essential plasmids, we compared the evolutionary dynamics of stable and unstable plasmids in two genetic host backgrounds.

**Fig 3:**
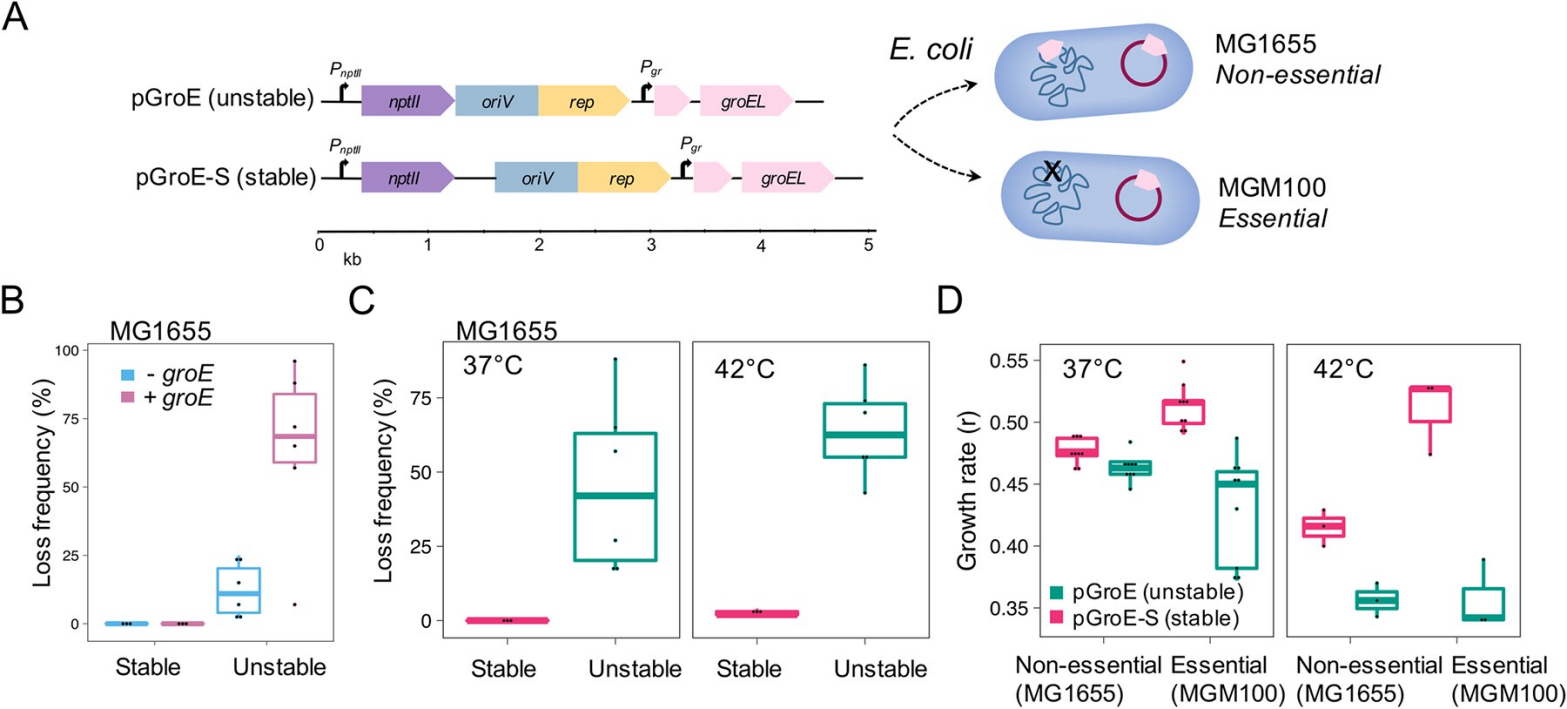
Characterization of plasmids encoding an essential gene. **A**, Genomic map of unstable pGroE and stable pGroE-S plasmids that were introduced into *E*. *coli* MG1655 (plasmid is non-essential) and *E*. *coli* MGM100 (plasmid is essential) hosts. **B**, Plasmid loss frequency of plasmids lacking (-) *groE* or encoding (+) *groE* in their genome in the host *E*. *coli* MG1655 (H_0_: *L*_*+*_*>L*_*-*_, *P* = 0.0247 using Wilcoxon test n = 6) **C**, Plasmid loss frequency of pGroE-S (stable) and pGroE (unstable) after incubation at 37°C or at 42°C (H_0_: *L*_*42*_*>L*_*37*_, *P* = 0.0091 using Wilcoxon test, n = 6). **D**, Growth rates of host strains *E*. *coli* MG1655 and *E*. *coli* MGM100 carrying pGroE (unstable) or pGroE-S (stable) after growth at 37°C and 42°C (H_0_: *Gr*_*42*_*>Gr*_*37*_, *P* = 0.051 using Wilcoxon test, n = 9).

Our unstable model plasmid pCON originated from the pBBR1 backbone that is widely spread and associated with antibiotic resistance [16,35]. The plasmid pCON encodes *nptII* that is constitutively expressed and confers resistance to kanamycin; it has a negligible effect on the host fitness and is characterized by an unstable inheritance in the population [16]. Previously, we showed that pCON instability is caused by transcription-replication conflicts of the resistance gene transcription and plasmid replication. A stable inheritance of pCON is provided when transcription is silenced or when both machineries are physically separated, i.e., by a plasmid size increase [16]. Our stable model plasmid pCON-S is a derivative of pCON that is characterized by a stable inheritance due to a short DNA insertion between the origin of replication and the *nptII* gene; similarly to pCON, it has a negligible effect on the host fitness [36]. We equipped the unstable (pCON) and stable (pCON-S) plasmids with the chromosomal *groE* (including its native promoter) resulting in pGroE and pGroE-S (Fig 3A). The plasmids pGroE and pGroE-S were introduced into two host strains: *E*. *coli* MG1655 (wt) and *E*. *coli* MGM100 (Fig 3A). Thus, *groE*-encoding plasmids are essential in *E*. *coli* MGM100 while they are non-essential in *E*. *coli* MG1655.

To characterize the effect of GroE on plasmid stability, we quantified the loss frequency of pGroE (unstable) and pGroE-S (stable). Our results show that the pGroE loss frequency was significantly higher in comparison to the loss frequency of the plasmid pCON lacking GroE in *E*. *coli* MG1655 (Fig 3B, *P* = 0.0247 using Wilcoxon test, n = 6). Similar to pCON-S, the plasmid pGroE-S was stably maintained in *E*. *coli* MG1655 in an overnight incubation (Fig 3B). Both plasmids were maintained in the host strain *E*. *coli* MGM100, where the plasmid is essential for host survival. When the medium was supplemented with arabinose–rendering the plasmid dispensable–we observed a plasmid loss frequency of 21±3% (SE, n = 6) for the unstable pGroE and no loss of the stable pGroE-S (n = 6; S19 Fig). These results are in line with the loss frequency of pGroE in *E*. *coli* MG1655, in which the plasmid is dispensable. Our results show that the introduction of a redundant *groE* copy on an unstable plasmid further destabilizes the plasmid maintenance and may lead to an increase in plasmid loss.

In addition to variation in the extent of plasmid-encoded gene essentiality, the observed plasmid loss may be related to the chaperonin function. The chaperonin GroE is part of the heat shock regulon in *E*. *coli* hence its expression level is upregulated during growth at high temperature (42°C) [37,38]. The expression level of plasmid-encoded genes depends not only on their transcriptional regulation but also on the plasmid copy number (PCN). Variation in PCN is thus comparable to gene amplification where fluctuations in PCN lead to variation in the expression level of plasmid-encoded genes, which may enable the host to rapidly adapt to changing environmental conditions [19,39]. Thus, under heat stress bacteria that carry plasmid-encoded *groE* are expected to have an advantage over strains with chromosomally encoded *groE* due to a higher GroE abundance in the cell. To evaluate whether heat stress had an effect on plasmid persistence, we measured the plasmid loss frequency after overnight incubation at 42°C. Our results show no measurable plasmid loss for both plasmids when the plasmid is essential (i.e., in *E*. *coli* MGM100). When the plasmid is not essential (i.e., in *E*. *coli* MG1655), the pGroE-S remained stable, while the pGroE loss frequency was slightly increased in comparison to pGroE loss at 37°C (Fig 3C). This observation is in line with reduced growth rate of MG1655 at 42°C in comparison to 37°C (Fig 3C, *P* = 0.0091 using Wilcoxon test). Thus, the redundant plasmid-encoded GroE does not confer an advantage in *E*. *coli* MG1655 at 42°C. Nonetheless, a comparison of the growth rate (r) between *E*. *coli* MGM100 pGroE-S and MG1655 pGroE-S at 42°C revealed a growth advantage of *E*. *coli* MGM100 pGroE-S over MG1655 pGroE-S (Fig 3D, *P* = 0.051 using Wilcoxon test) and over the plasmid-free wildtype (r_wt_ = 0.42±0.01, SE, n = 9). The growth advantage observed for expression *E*. *coli* MGM100 pGroE-S likely stems from differences in *groE* expression dynamics when it is expressed solely from the plasmid locus. Additionally, the growth rate of both strains carrying the unstable pGroE was decreased (Fig 3D, *P* = 0.0007 using Wilcoxon test). The reduced growth rate of *E*. *coli* MGM100 carrying the unstable pGroE is well explained by the occurrence of cell death following plasmid loss (S20 Fig).

We conclude that the presence of a redundant essential gene on an unstable plasmid may destabilize plasmid maintenance in the host regardless of growth conditions. The maintenance of stable plasmids can remain, however, unchanged in the presence of an essential gene. Furthermore, variation of the plasmid-encoded gene expression level due to fluctuations in the plasmid copy number may confer an advantage to the host under specific environmental conditions.

### Essential plasmids persist over time regardless of their stability

Constant positive selection for a beneficial gene is expected to result in gene transfer from the plasmid to the chromosome and consequently plasmid loss [40,41]. To test the effect of plasmid essentiality on plasmid evolution, we conducted an evolution experiment comparing both unstable (pGroE) and stable (pGroE-S) plasmids in host backgrounds where the plasmids are essential or non-essential. The experiment was performed with nine replicates of all plasmid and host combinations for approximately 320 generations in a serial batch transfer approach.

**Fig 4:**
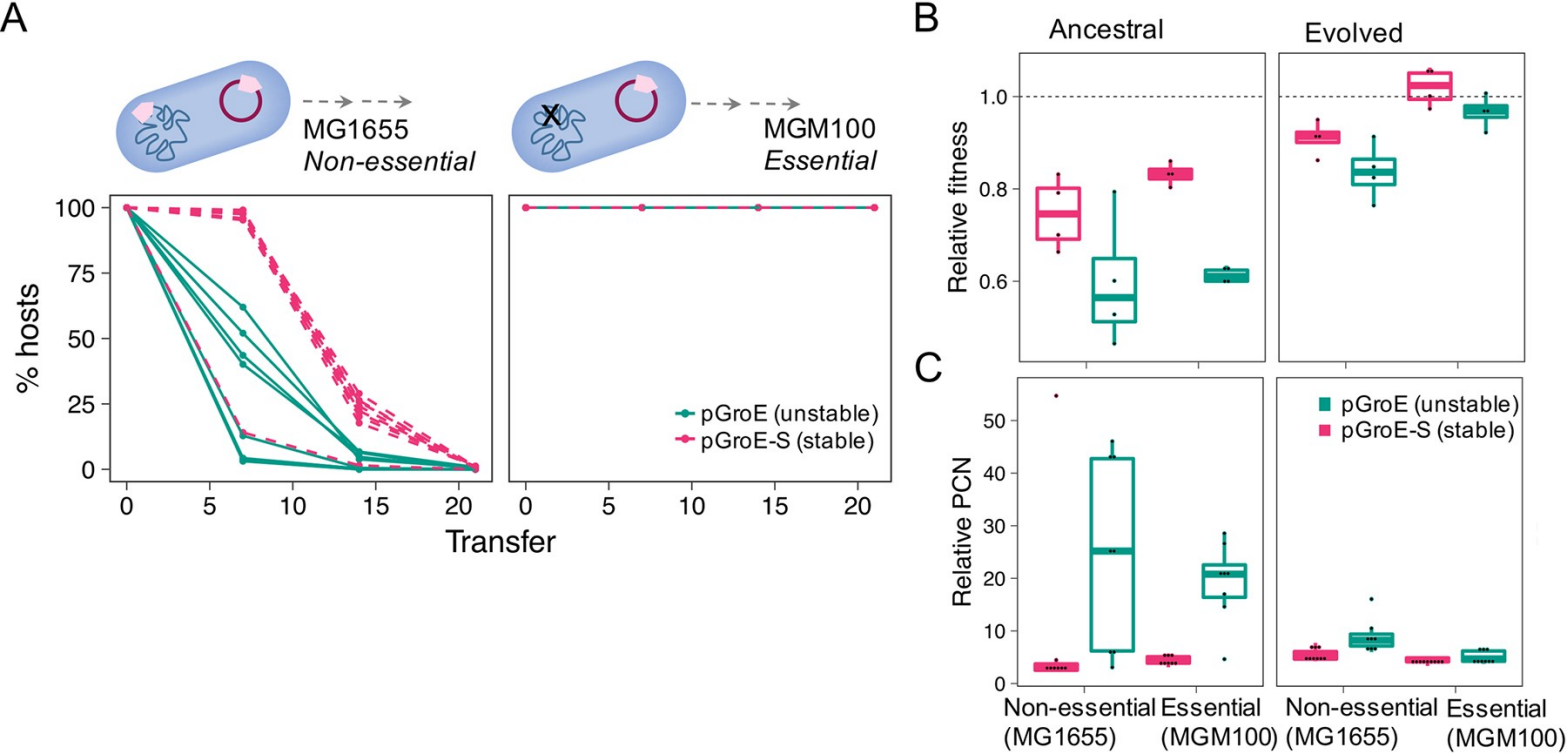
Evolution of plasmids encoding an essential gene. **A,** Evolution experiment of pGroE (unstable) and pGroE-S (stable) in the host strains *E*. *coli* MG1655 (non-essential, left) and MGM100 (essential, right). **B**, Relative fitness of plasmid-carrying ancestral and evolved populations. Pairwise competition experiments between the plasmid-carrying strains and tagged wild-type (Tm^r^). A significant negative fitness effect could be observed for all ancestral populations (*w*: 0.69, *P* = 1.526x10^-5^ using Wilcoxon test, n = 4 per population) while the fitness of evolved populations increased compared to the ancestral state (*w*: 0.93, *P* = 9.05x10^-7^ using Wilcoxon test, n = 4 per population). **C**, Plasmid copy number (PCN) of plasmid-carrying ancestral and evolved populations. The median pGroE PCN is significantly different between the ancestral and evolved populations (*P*_*MG1655*_ = 0.03, *P*_*MGM100*_ = 0.0016, using Wilcoxon test, n = 9 per population), with the PCN being lower in the evolved populations in both hosts.

The results of the evolution experiment reveal that when the plasmids were essential for host survival, they were maintained over time regardless of their stability (Fig 4A). Sequencing a sample of the evolved populations, we did not detect evidence for *groE* transfer to the chromosome or mutations in the inducible P_BAD_-*groE* promoter (S5 Table). When the plasmid was non-essential to the host (i.e., in *E*. *coli* MG1655), the highly unstable pGroE was lost rapidly while the stable pGroE-S decreased in frequency after several transfers (Fig 4A). Thus, non-essential plasmids were lost regardless of their initial stability in the host (i.e., stable inheritance). To test whether plasmid loss in our experiment led to plasmid extinction, we exposed the evolved populations to antibiotics (kanamycin 25 μg/ml; S21 Fig), which was then followed by serial transfer under non-selective conditions (i.e., without antibiotics). This experiment showed that none of the plasmids went extinct over time. In addition, we observed a similar plasmid loss pattern, where the unstable plasmid was gradually lost from the population and the stable plasmid was maintained at a higher frequency (S21 Fig). Our results thus suggest that the decreased frequency of plasmid carrying hosts is not due to plasmid instability, rather is a result of host fitness differences between plasmid-carrying and plasmid-free cells.

To evaluate the relative fitness of the plasmid-carrying populations, we conducted competition experiments of the ancestral and evolved populations against the plasmid-free wildtype strain. Indeed, we observed a strong negative fitness effect of all *groE*-plasmids on host fitness (Fig 4B; *w*: 0.697, *P* = 1.52x10^-5^ using Wilcoxon test, n = 4 per population). Nonetheless, the negative impact on fitness decreased over time and the evolved populations revealed a higher relative fitness than their ancestors (Fig 4B, *w*: 0.93, *P* = 9.05x10^-7^ using Wilcoxon test, n = 4 per population). A reduction in the plasmid effect on the host fitness might have been associated with a decrease in PCN over time. Indeed, this is the case for the unstable plasmids in both host strains where the average PCN decreased about 10-fold (Fig 4C). Previously, we showed that a reduction of PCN for pCON plasmids is often a result of reduction in the plasmid tendency to form multimers (Wein et al., 2019; 2020). Moreover, the variance of pGroE PCN (in MG1655) among replicate populations decreased during the experiment (ancestral CV_pGroE_: 0.73, evolved CV_pGroE_: 0.35), and so did the loss frequency (S22 Fig), a result that is akin to the loss of plasmid multimers during the evolution experiment (as in [16,36]). Since we did not detect any high frequency chromosomal variants in the evolved populations (S5 Table; as previously observed for antibiotic resistance plasmids [42]), we conclude that the decrease in PCN is likely due to an efficient resolution of plasmid multimers in the evolved population.

Our results show that plasmid essentiality due to complementation of the chaperonin function leads to plasmid persistence that is accompanied by an increase in overall fitness of the evolved populations. Nonetheless, carrying a redundant chromosomal gene proved here to be disadvantageous for plasmid persistence regardless of plasmid stability. Consequently, we conclude that plasmids encoding an essential gene may have a higher chance to persist in the population over longer time scale if a chromosomal gene copy is either lacking or functionally divergent.

## Discussion

The acquisition of an additional copy of single-copy chromosomal genes has been shown to be often deleterious, hence sensitivity to dose effect has been suggested as a barrier to gene acquisition by lateral gene transfer [23]. Newly acquired genes are often initially disconnected from the organisms’ metabolic network and their integration may take a long time [43]. Thus, additional copies of core essential genes can disrupt the cell physiology through protein dosage alterations that change metabolic fluxes (e.g., [44]). Regulatory networks in prokaryotes have evolved to respond to feedback loops that include monitoring of transcription level and substrate concentrations. Hence, the acquisition of a redundant gene copy may be deleterious due to indirect effect on the organism metabolism and cellular processes [22,45]. Notwithstanding, plasmid-mediated dose effect is not always deleterious to the host and may depend on the protein sequence characteristics and interactions with other proteins in the cell [46]. Our results for the chaperonin-encoding plasmid reveal that functional redundancy of an essential gene following plasmid acquisition can be highly disadvantageous for host fitness. In our experiment, the GroE abundance is associated with the PCN, hence the reduction in fitness effect of the plasmid in the evolved populations is likely explained by the decrease in PCN that is coupled with a decrease in GroE dose within the cell. An elevated expression level of the chaperonin has been shown to be detrimental for *E*. *coli* fitness under optimal growth conditions [47,48]. The fitness cost of GroE overexpression has been suggested to stem from the increased energetic investment in protein folding due to the high chaperonin availability [49]. The plasmid-mediated increase in GroE abundance may furthermore hamper the chaperonin assembly in the cell. Plasmids that gain a redundant essential gene are thus at risk of rapid loss and eventually extinction.

Based on our results, we suggest that the evolution of plasmids encoding essential genes is conditioned by either non-functionalization (or loss) of the chromosomal copy or its functional divergence, e.g., via neo-functionalization or sub-functionalization due to modification of the transcriptional regime. This constraint provides an explanation for the rarity of essential genes in *Escherichia* plasmids. Notably, two of the plasmid-encoded essential genes we report here–*ssb* and *groE*–seem to have undergone a sub-functionalization as part of the plasmid life cycle such that their function is no longer essential for the host. In other words, those essential genes were acquired by the plasmid and repurposed into essential plasmid genes that are required for persistence in the host. The *Escherichia* plasmids encoding *ssb* or *groE* are thus carrying homologs to essential genes but should not be considered essential plasmids. Hence, we predict that essential genes are rarely found on plasmids in other prokaryotic taxa and natural environments. The detrimental effect of essential gene acquisition on plasmid stability constitutes a barrier for plasmid-mediated lateral transfer of essential genes. Nonetheless, we cannot exclude that plasmids encoding an essential gene may become advantageous under specific environmental conditions. Our results show that the translocation of *groE* to a plasmid increased host fitness during growth at high temperature where *groE* expression is naturally upregulated [37,38]. Indeed, *groE* may be found on plasmids in nature as in the facultative plant symbiont *Sinorhizobium meliloti*, in which two out of five functional *groE* copies are encoded on the plasmid pSymA and one copy is encoded on the plasmid pSymB [50]. The chromosomal *groE1* copy was shown to encode the major housekeeping chaperonin function, yet it could be functionally complemented by the pSymA-encoded *groE2* [51]. The reason why multiple *groE* copies are retained in *S*. *meliloti* remains unknown. It is tenable to speculate that the facultative symbiotic lifestyle of *S*. *meliloti* entailed a sub-functionalization of the *groE* copies. An example for the translocation of an essential gene from the chromosome to a plasmid was reported in bacterial symbionts of lice that inhabit human, chimpanzee and gorilla (*Riesia* sp.). In these organisms, the B5 vitamin biosynthesis pathway is encoded on a small plasmid that is vital for the bacteria-louse symbiosis. In contrast, symbionts of lice that inhabit old world monkeys (*Puchtella* sp.) are lacking the plasmid and the B5 vitamin pathway is chromosomally encoded [52]. The phylogenetic relations of the two symbionts indicate that the chromosomal B5 copy is ancient and the gene translocation to the plasmid occurred during the lice adaptation to inhabit new-world monkeys [52]. We hypothesize that the fixation of the gene translocation on the plasmid may be related to the symbiotic *Riesia* lifestyle; a retention of the plasmid encoded gene copy may be advantageous under conditions that require a flexible gene expression level, which can be regulated rapidly by transient changes in plasmid copy number [39].

Considering the two possible routes for the evolution of essential genes on plasmids–our study supplies evidence that the *essentiality first* route is the more likely scenario. To better understand the difference between the two routes we shift our perspective to consider the fitness of the plasmid. Plasmid loss in our experimental system stems from two main reasons: plasmid instability of the unstable pGroE or fitness disadvantage of the stable pGroE-S-hosts; indeed, incomplete segregation and negative fitness effects are known causes of plasmid loss (e.g., [53,54]). The comparison between the loss dynamics of the unstable and stable plasmids (Fig 4A) indicates that plasmid loss due to intracellular processes–namely plasmid instability–may be more rapid in comparison to plasmid loss due to processes at the population level–where plasmid-hosts are outcompeted by non-hosts. Our results indicate that the acquisition of an essential chromosomal gene by a plasmid leads to a significant reduction in plasmid fitness, even when the plasmid was highly stable prior to the essential gene acquisition (as in the *stability first* route). Considering the constraint on the presence of chromosomal essential genes on plasmids, most plasmids are unlikely to be essential for their host.

## Methods

### Computational genomic analysis

All 599 completely sequenced genomes of *Escherichia* genus strains were downloaded from NCBI (version 2018) and analyzed as described in [36]. Briefly, plasmid-encoded protein coding genes were clustered into homologous protein families. Sequence similarity of plasmid-encoded protein sequences was assessed from the results of reciprocal best BLAST hits (rBBHs) applying a threshold of E-value ≤ 1×10^−5^ (using BLAST version 2.6.0+ [55]). Pairs of rBBHs were further compared by global alignment with needle (version 6.6.0.0; EMBOSS package [56]. Sequence pairs with ≥50% identical amino acids were clustered into protein families using the Markov clustering algorithm (MCL) (version 12–135) with the default parameters [57]. To identify chromosome-encoded homologs of plasmid genes, the sequences of all plasmid protein families were blasted against all the chromosome proteins using BLAST with a threshold of E-value ≤ 1×10^−10^. The resulting chromosomal hits were further compared by global alignment using needle; chromosome-encoded protein sequences were considered homologs using a threshold of ≥40% identical amino acids. AMR protein families were identified using Resistance Gene Identifier (version 5.1.0), with CARD database (version 3.0.7) [58]. Plasmid mobility was inferred depending on the presence of the Mob relaxase and T4SS encoding genes in the plasmid genomes (as listed in [59]). Plasmids were classified as mobilizable if they encode a Mob protein and as conjugative if they have at least 15 *tra* genes. The dataset of essential genes in *E*. *coli* includes 394 protein-coding genes included in the essential genome of *E*. *coli* K-12 [25]. These genes were identified manually in the genome of *E*. *coli* K-12 substr. MG1655 according to their gene symbol. This dataset was supplemented with additional 110 protein-coding genes identified as essential in a large-scale study that included multiple *E*. *coli* isolates and three growth media [18], conditions that the gene was essential in all 18 tested isolates in at least one of the tested media (LB, GMM, M9). These genes were identified manually in the genome of *E*. *coli* str. K-12 substr. MG1655 according to the gene symbol and locus tag. The final set included 504 protein-coding essential genes (listed in S2 Table). The protein sequences of chromosome-encoded essential genes and their plasmid homologs were aligned using MAFFT (version v7.123b) [60]. Maximum likelihood trees were reconstructed using IQ-Tree (version 1.5.5) [61] with LG model. The 17 alignments and trees are available as supplementary data. Trees were rooted using the midpoint criterion. The topology of trees comprising a deep plasmid-chromosome split and putative LGT events was further tested with IQ-TREE as following: a constrained tree topology assuming vertical evolution only (i.e., assuming a deep chromosome-plasmid split) was reconstructed; The likelihood of the resulting constrained topology was compared to the unconstrained topology (i.e., with the LGT event) using the approximately unbiased (AU) topology test. A rejection of the constrained topology thus supports the LGT inference. Codon-aware alignments were produced using PAL2NAL program [62]. For trees with a split formed by homologs on different replicons, selective strength on different branches were calculated using HyPhy-RELAX (version 2.5.15) [63]. The resulting trees were visualized using iTOL v5 (https://itol.embl.de/) [64]. Conserved syntenic blocks (i.e., gene order) were identified using CSBFinder-S [65] allowing ≥ 3 insertions.

### Bacterial strains, plasmids and culture conditions

The strain *Escherichia coli* K-12 MG1655 was used as the model organism in all experiments (DSM No. 18039, German Collection of Microorganisms and Cell Cultures, DSMZ). In addition, we used the *E*. *coli* strain MGM100 that encodes the *groE* operon under an inducible P_BAD_ promoter [34]. For the purpose of competition experiments, *E*. *coli* MG1655 equipped with a chromosomal mini-Tn*7* insertion (attTn7:miniTn*7(dhfrII)* [16]) conferring resistance to trimethoprim (Tm^r^) was used. The strain *E*. *coli* DH5α was used during plasmid construction. All *E*. *coli* strains were routinely grown at 37°C in Luria Bertani (LB- Lennox) medium at 250 rpm shaking. 1% Arabinose was absent from the growth media, unless when stated otherwise. For molecular cloning and documentation purpose, the plasmid carrying strains were grown in LB supplemented with kanamycin (25 μg per ml).

The plasmids in this study are derived from the plasmids pCON and pCON-S that were constructed previously [16,36]. Their backbone is comprised of a pBBR1 origin (*rep* and *oriV* [35]) and they carry the antibiotic resistance gene *nptII* encoding for a neomycin phosphotransferase that confers resistance to kanamycin (including the natural Tn*5* promoter [66]). The plasmids pGroE and pGroE-S were built by PCR amplification of pCON and pCON-S (for primers see S6 Table). The chromosomal operon *groE*, including its native promoter, was amplified from the genome of *E*. *coli* MG1655 by PCR using the primers groE_for/rev (S6 Table). Subsequently, the plasmids were assembled using the NEBuilder® protocol (New England Biolabs). The plasmid-derived GroE (WP_004201172.1, WP_004201176.1) was custom synthesized by Genscript and comprised the sequence of the strain *E*. *coli* 6409 (NZ_CP020056.1; Fig 2C). The sequence was introduced into pCON-S by PCR amplification (for primers see S6 Table) and NEBuilder® asssembly. All plasmids were extracted using the GeneJET Plasmid Miniprep Kit (Thermo Fisher Scientific) and DNA concentrations were measured using a NanoDrop (Thermo Fisher Scientific).

### Fitness experiments

The relative fitness (*w*) [67] of plasmid-carrying versus ancestral plasmid-free strain (wt) was estimated by pairwise competition experiments, with four replicates per plasmid type. All competition experiments were initiated with a 1:1 mixture of 1:100 diluted plasmid-carrying and ancestral strain (*E*. *coli* MG1655 Tm^r^ [16]) from overnight cultures, in a total volume of 1 ml of non-selective LB medium. The relative fitness of plasmid host strains was calculated by evaluating cell counts at time points 0 h and 24 h. The strains were distinguished through plating on non-selective (LB) and selective media (LB supplemented with trimethoprim 150 μg per ml and LB supplemented with kanamycin 25 μg per ml). The chromosomal integration (Tm^r^) as well as the plasmid pCON have no measurable impact on the fitness of *E*. *coli* MG1655 (previously shown in [16]).

### Evolution experiment

Evolution experiments were conducted with plasmid-carrying strains under non-selective conditions. On the onset of the experiment, the host strains were plated on selective media to ensure plasmid carriage (kanamycin, 25 μg per ml). The experiments were founded by nine replicate colonies of each plasmid type which were inoculated in LB medium at 37°C constant shaking. After overnight growth all cultures were diluted 1:100,000 and transferred into 96-deep-well plates in a total volume of 1 ml. The diluted cultures were incubated at 37°C with constant shaking. The populations were transferred every 24 h by diluting the cultures 1:100,000 and the serial transfer was repeated over a total of 23 transfers for pGroE/pGroE-S populations. The number of generations was routinely measured by evaluating the cell number through plating directly after the dilution and before the next subsequent transfer. We observed a total number of ~320 generations for the pGroE/pGroE-S populations. During the evolution experiment, the frequency of plasmids in the population was estimated from the proportion of hosts, which was determined by replica plating (Lederberg and Lederberg 1952) every 7 transfers. Briefly, this was performed by first serially diluting the grown cultures followed by plating of ~500 cells on non-selective LB media. The number of plated cells was increased with decreasing plasmid frequency up to ~1000 cells. The plated populations were incubated for overnight growth. Colonies were counted and replicated using velvet cloth on selective media (LB supplemented with kanamycin 25 μg per ml). Colonies growing on the selective media were counted as plasmid hosts (for a detailed protocol see [68]).

To test for plasmid extinction and plasmid stability evolution, the evolved populations were transferred into a 96-deep-well plate containing selective media (LB supplemented with kanamycin, 25 μg per ml) and incubated for overnight growth (12 h). Thereafter, the cultures were transferred into non-selective conditions for a follow-up evolution experiment. Plasmid host frequency was monitored via replica plating.

### Plasmid loss frequency assays

The plasmid loss frequency was estimated from the frequency of plasmid free cells occurring during overnight growth in non-selective media. To determine the loss frequency, cultures were inoculated from single colonies grown on selective media to ensure plasmid carriage. After 12 h growth in 37°C (approximately 8.5 generations), the cultures were serially diluted and plated on non-selective LB media. After overnight incubation the plates were replicated. Following overnight growth, colonies grow on replicated plates were counted as plasmid carrying. The loss frequency was calculated from plasmid-free cells (not resistant) and the total number of colonies tested.

### Plasmid copy number determination

Plasmid copy number (PCN) was determined using quantitative real time PCR (qPCR) (as described in [16]). Bacterial cells were lysed by 10 min incubation at 98°C directly followed by 10 min at -20°C. The qPCR was conducted with primers targeting the chromosome and the plasmid DNA. The chromosomal primers were complementary to the *idnT* gene of *E*. *coli* (q_idnT_F/R) and the plasmid primers targeted the *nptII* gene (q_nptII_F/R). The qPCR reactions were conducted in volume of 10 μl containing 1x iTaq Universal SYBR Green Supermix (Bio-Rad Laboratories), 100 nM of each primer (final concentration) and 1 μl sample. All qPCR reactions including positive and non-template controls were performed in technical replicates on a Real-Time PCR Detection System (Bio-Rad) using: 95°C for 3 min, and 40 cycles of 10 s at 95°C and 1 min at 59°C cycling conditions for all reactions. The ratio between the number of plasmid amplicons and chromosome amplicons is defined as the plasmid copy number (comparative C_T_ (ΔΔC_T_) method) and was calculated while considering the amplification efficiencies of both primer pairs.

### Population growth dynamics

The growth dynamics of *E*. *coli* populations carrying different plasmids were measured by determining the optical density at 600 nm (OD_600_) using a photo-spectrometer (Thermo Fisher Scientific) during growth at 37°C or 42°C for 24 h. Thereafter, the R package *growthcurver* [69], was used to fit a logistic growth model to the growth dynamics and estimate the growth parameters including the growth rate (r).

### Bacterial viability assay

Bacterial viability was evaluated by staining *E*. *coli* cells with Propidium iodide (PI, Sigma). PI enters only compromised bacterial membranes and is therefore an indicator of membrane integrity (i.e., does not enter alive cells) and only stains dead cells. *E*. *coli* populations were inoculated in liquid media and the cultures were sampled after two hours (log cells) and ten hours (stationary cells) of growth (3 or 4 replicates per strain). The cells were washed in PBS (20 μl) and incubated with PI at a final concentration of 30 μM for 10 min in the dark at room temperature. Thereafter, 10 μl of the mixture were transferred to an agar-coated slide and the cells were visualized with an epifluorescence microscope (Zeiss Axio Imager 2, Plan-Apochromat 63×/1.40 Oil DIC M27 objective). For counting purposes at least 10 images were taken per sample at random locations. The cell number of stained versus non-stained cells were evaluated using the software ImageJ.

### Sequencing analysis

Population sequencing of the ancestral and evolved populations (one or two per treatment) was used to detect genetic variants occurring either on the plasmid or the host chromosomes. Prior to the sequencing, cultures were grown in antibiotics to ensure plasmid presence (see S20 Fig). Total DNA was isolated from 1 ml culture using the Wizard Genomic DNA Purification Kit (Promega). Concentration and quality of the extracted plasmid and genomic DNA was assessed using the NanoDrop (Thermo Fisher Scientific) and Qubit (Invitrogen by Life Technologies). The sample libraries for Illumina sequencing were prepared using the Nextera Flex library kit (Illumina, Inc) and sequencing was performed with paired-end reads on the MiSeq platform (Illumina, Inc).

Sequencing reads were trimmed to remove Illumina specific adaptors and low quality bases using the program Trimmomatic v.0.35 [70] (parameters: ILLUMINACLIP:NexteraPE-PE.fa:2:30:10 CROP:250 HEADCROP:5 LEADING:20 TRAILING:20 SLIDINGWINDOW:4:20 MINLEN:36). As the reference we joined the *E*. *coli* MG1655 (GenBank acc. no. NC_000913.3) genome with the plasmid sequences (created in SnapGene software v.2.4 (GLS Biotech)). The sequencing reads were mapped to the reference genomes using BWA-MEM v.0.7.5a-r405 [71]. Mapping statistics were retrieved using BAMStats v.1.25 (https://sourceforge.net/projects/bamstats/files/). Subsequent indexing and local realignment of sequencing reads were performed using PICARD tools, SAMtools v.0.1.19 [72] and GATK v.3.6 [73] retaining only paired mapped reads with a minimum mapping quality of 20. SNPs were called using LoFreq v.2.1.2 [74].

### Statistical analysis

All statistical tests and data analysis were performed in R version 3.5.1.

## Supporting information

S1 Fig An empirical cumulative distribution function (CDF) describing the number of isolates per plasmid protein family in which a chromosomal homolog to a plasmid protein family was found. An inlay plot (in red dashed line) shows an enlargement of the top left corner. The distribution shows that 81% plasmid protein families have no chromosomal homolog in the same isolate. The remaining 19% plasmid protein families have a chromosomal homolog in at least one isolate.
(TIF)

S2 Fig Phylogeny of ATP-dependent metalloprotease FtsH homologs in *Escherichia* strains. Isolate names are colored according to gene location with red for plasmids and blue for chromosomes.
(TIF)

S3 Fig Phylogeny of DNA replication protein DnaC homologs in *Escherichia* strains. Isolate names are colored according to gene location with red for plasmids and blue for chromosomes.
(TIF)

S4 Fig Phylogeny of methionine—tRNA ligase MetG homologs in *Escherichia* strains. Isolate names are colored according to gene location with red for plasmids and blue for chromosomes. MetG in *Escherichia coli* isolate 2014C-3307 is found only on a plasmid (i.e., it has no chromosomal homolog).
(TIF)

S5 Fig Phylogeny of glutamine—tRNA ligase GlnS homologs in *Escherichia* strains. Isolate names are colored according to gene location with red for plasmids and blue for chromosomes.
(TIF)

S6 Fig Phylogeny of gamma-glutamylphosphate reductase ProA homologs in *Escherichia* strains. Isolate names are colored according to gene location with red for plasmids and blue for chromosomes.
(TIF)

S7 Fig Phylogeny of gamma-glutamate kinase ProB homologs in *Escherichia* strains. Isolate names are colored according to gene location with red for plasmids and blue for chromosomes.
(TIF)

S8 Fig Phylogeny of aromatic acid decarboxylase UbiX homologs in *Escherichia* strains. Isolate names are colored according to gene location with red for plasmids and blue for chromosomes.
(TIF)

S9 Fig Phylogeny of ribonucleotide-diphosphate reductase subunit beta NrdB homologs in *Escherichia* strains. Isolate names are colored according to gene location with red for plasmids and blue for chromosomes.
(TIF)

S10 Fig Phylogeny of ribonucleoside-diphosphate reductase subunit alpha NrdA homologs in *Escherichia* strains. Isolate names are colored according to gene location with red for plasmids and blue for chromosomes.
(TIF)

S11 Fig Phylogeny of peptide deformylase Def homologs in *Escherichia* strains. Isolate names are colored according to gene location with red for plasmids and blue for chromosomes.
(TIF)

S12 Fig Phylogeny of ketol-acid reductoisomerase LivC homologs in *Escherichia* strains. Isolate names are colored according to gene location with red for plasmids and blue for chromosomes.
(TIF)

S13 Fig Phylogeny of lipoprotein-releasing ABC transporter permease subunit LolC homologs in *Escherichia* strains. Isolate names are colored according to gene location with red for plasmids and blue for chromosomes.
(TIF)

S14 Fig Phylogeny of D-arabinose 5-phosphate isomerase KdsD homologs in *Escherichia* strains. Isolate names are colored according to gene location with red for plasmids and blue for chromosomes.
(TIF)

S15 Fig Branch specific parameters of relaxation of selection inferred for GroEL phylogeny using HyPhy-RELAX. Branches are colored according to the selection intensity parameter k, ranging between brown for k<1 (relaxed selection) and green for k>1 (intensified selection). Note that branch length information is excluded in this figure. Isolate names are colored according to gene location with red for plasmids and blue for chromosomes.
(TIF)

S16 Fig Branch specific relaxation parameters are inferred for NrdA phylogeny using HyPhy-RELAX. Branches are colored according to the selection intensity parameter k, ranging between brown for k<1 (relaxed selection) and green for k>1 (intensified selection). Note that branch length information is excluded in this figure. Isolate names are colored according to gene location with red for plasmids and blue for chromosomes.
(TIF)

S17 Fig Phylogeny of replicative DNA helicase DnaB homologs in *Escherichia* strains. Isolate names are colored according to gene location with red for plasmids and blue for chromosomes.
(TIF)

S18 Fig Relative Fitness of *E. coli* MGM100 in comparison to *E. coli* MG1655 (n = 4). (TIFF)

S19 Fig Plasmid loss of pGroE and pGroE-S in *E. coli* MGM100 after overnight growth in the presence of 1% arabinose (n = 6). (TIFF)

S20 Fig Quantification of the number of dead cells along the growth phase of *E. coli* MG1655 and MGM100 populations. The number of dead cells was evaluated in the log phase and in the stationary growth phase (n = 4). (TIFF)

S21 Fig Plasmid frequency following evolution experiment. Plasmid carrying populations were exposed to a transfer in antibiotics (kanamycin 25 μg/ml).
(TIFF)

S22 Fig Plasmid loss frequency of evolved plasmids pGroE-S and pGroE in the host strain *E. coli* MG1655. The plasmid loss was measured after overnight incubation (n = 6).
(TIFF)

S1 Table List of 599 Escherichia strains used in the comparative genomic analysis. A total of 13 contigs were excluded from the analysis either because they better matched phage genomes, or were suspected as mis-assembly artifacts (i.e., suspected as chromosome sequences rather than plasmid sequences. Accession number are listed in the table).
(XLSX)

S2 Table List of 504 essential protein-coding genes in *Escherichia coli* used in this study. (XLSX)

S3 Table Conserved neighborhood of the single-strand DNA binding protein (Ssb) encoded on plasmids. The conserved genomic neighborhood comprises five genes including: *ssb* encoding single-strand DNA binding protein, *parB* encoding for a type II partitioning system as well as *psiA* and *psiB* that are known as inhibitors of the SOS response.
(XLSX)

S4 Table Conserved neighborhood of the chaperonin complex (GroEL/S) encoded on plasmids. The conserved genomic neighborhood comprises 7–8 genes that include the chaperonin complex (*groEL* and *groES*), *bla*_NDM-1_, *ble* (bleomycin binding protein Ble-MBL), a gene encoding a phosphoribosylanthranilate isomerase, a gene encoding twin-arginine translocation (TAT) pathway signal sequence domain protein and *cutA* (divalent-cation tolerance protein). In six out of ten instances the conserved neighborhood includes a gene encoding for a IS*91* family transposase.
(XLSX)

S5 Table Sequencing results of plasmid-carrying strains. Sequenced samples include ancestral populations as well as evolved strains carrying the plasmid pGroE or pGroE-S.
(TIFF)

S6 Table Oligonucleotides used in this study. (TIFF)

S1 Data Alignments and trees of the essential gene families. (GZ)
