# Supplementary figures and images for: Essential gene acquisition destabilizes plasmid inheritance
Source: PLoS Genet. 2021 Jul 12;17(7):e1009656. doi: 10.1371/journal.pgen.1009656 (PMC8297927; doi:10.1371/journal.pgen.1009656)

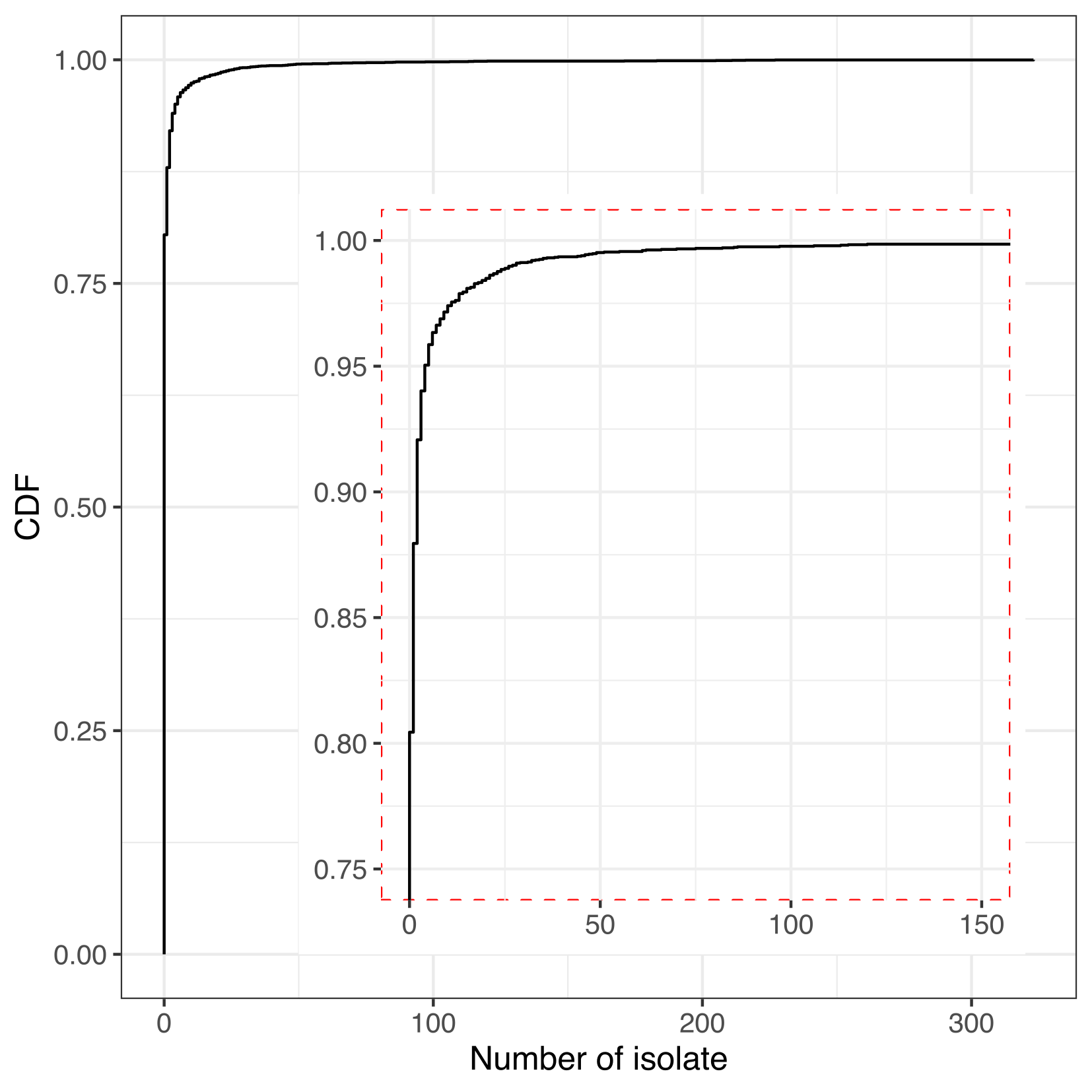

Supplement: S1 Fig — An inlay plot (in red dashed line) shows an enlargement of the top left corner. The distribution shows that 81% plasmid protein families have no chromosomal homolog in the same isolate. The remaining 19% plasmid protein families have a chromosomal homolog in at least one isolate. (TIF) [file pgen.1009656.s001.tif]

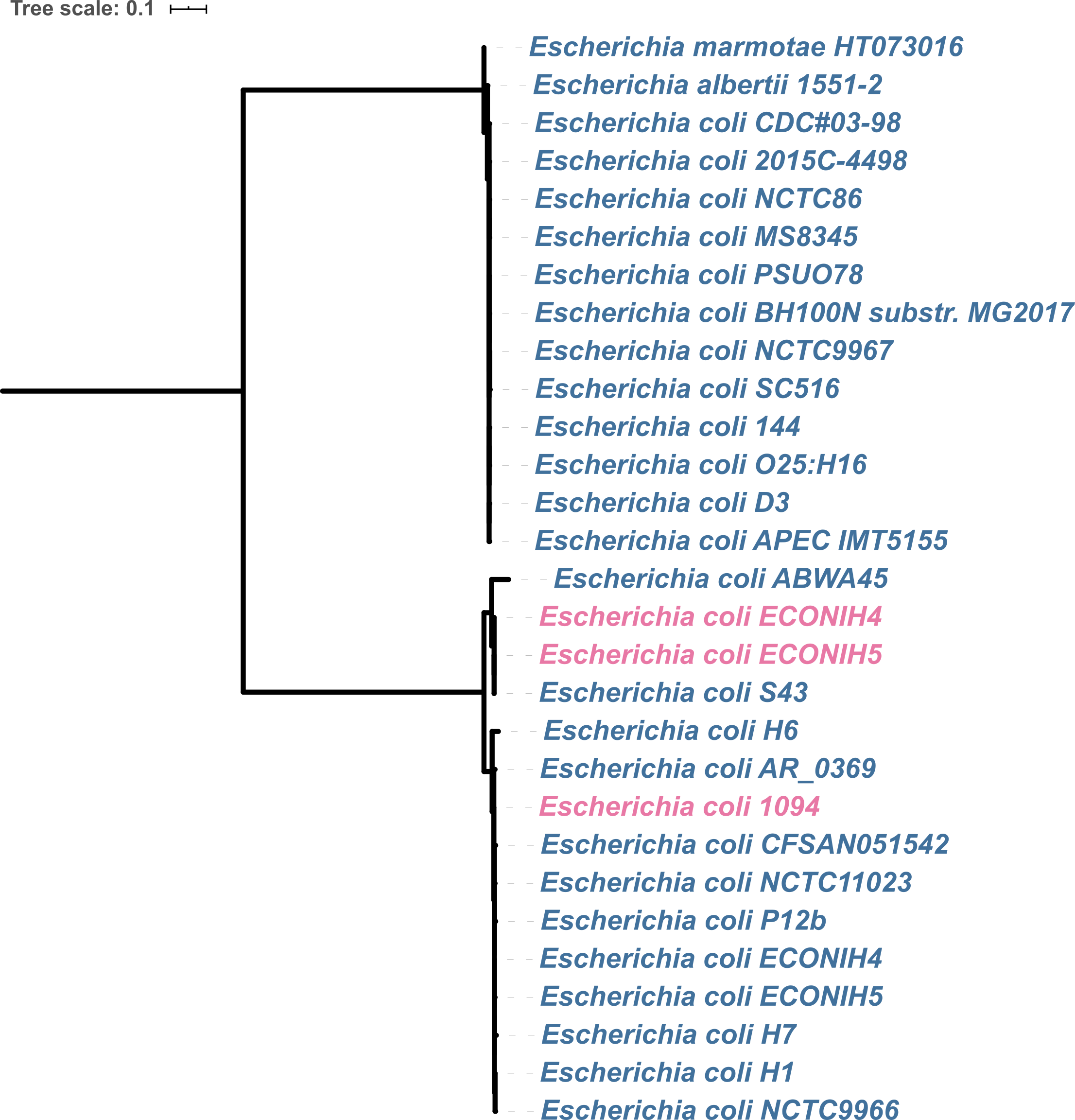

Supplement: S2 Fig — Isolate names are colored according to gene location with red for plasmids and blue for chromosomes. (TIF) [file pgen.1009656.s002.tif]

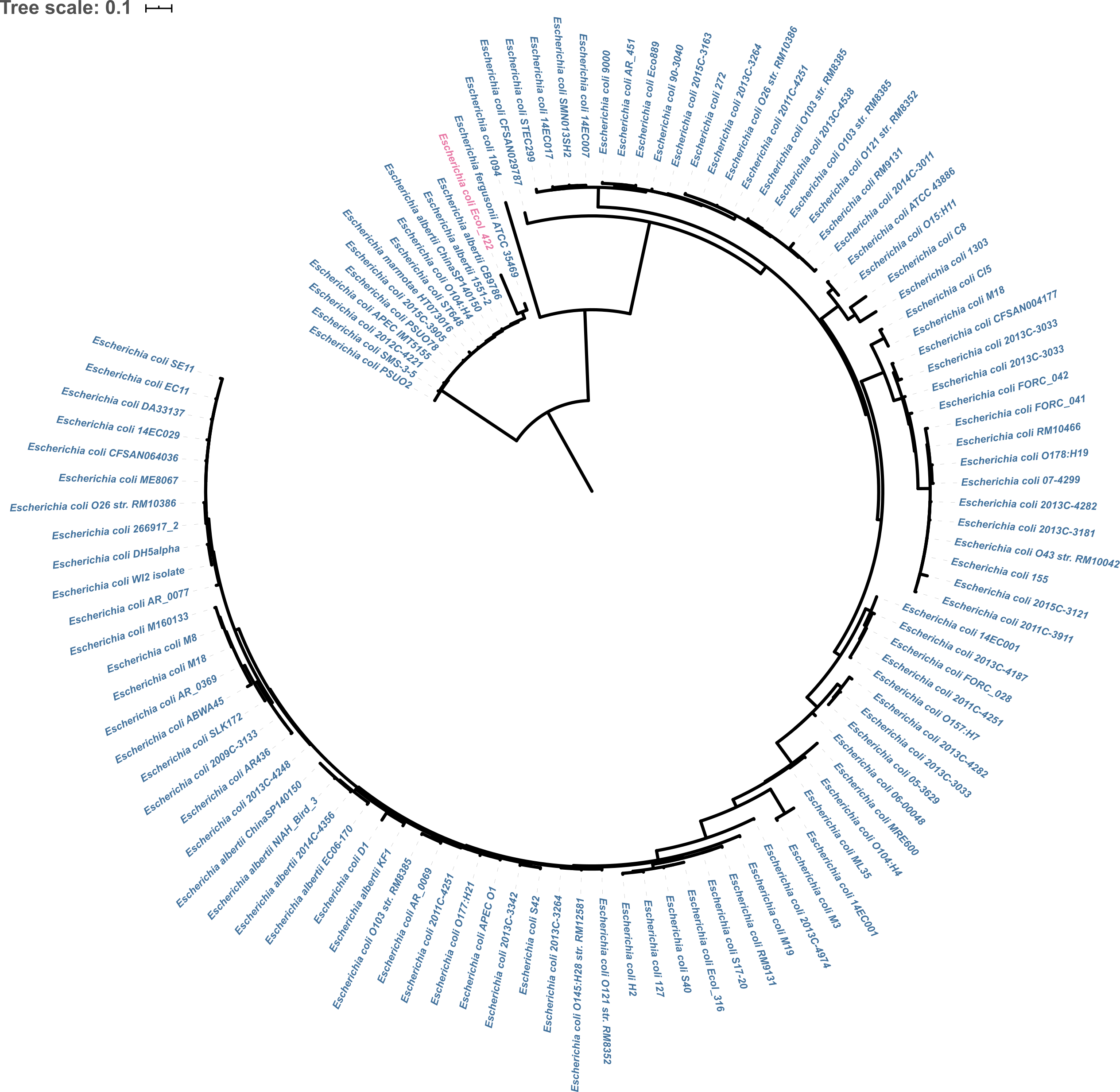

Supplement: S3 Fig — Isolate names are colored according to gene location with red for plasmids and blue for chromosomes. (TIF) [file pgen.1009656.s003.tif]

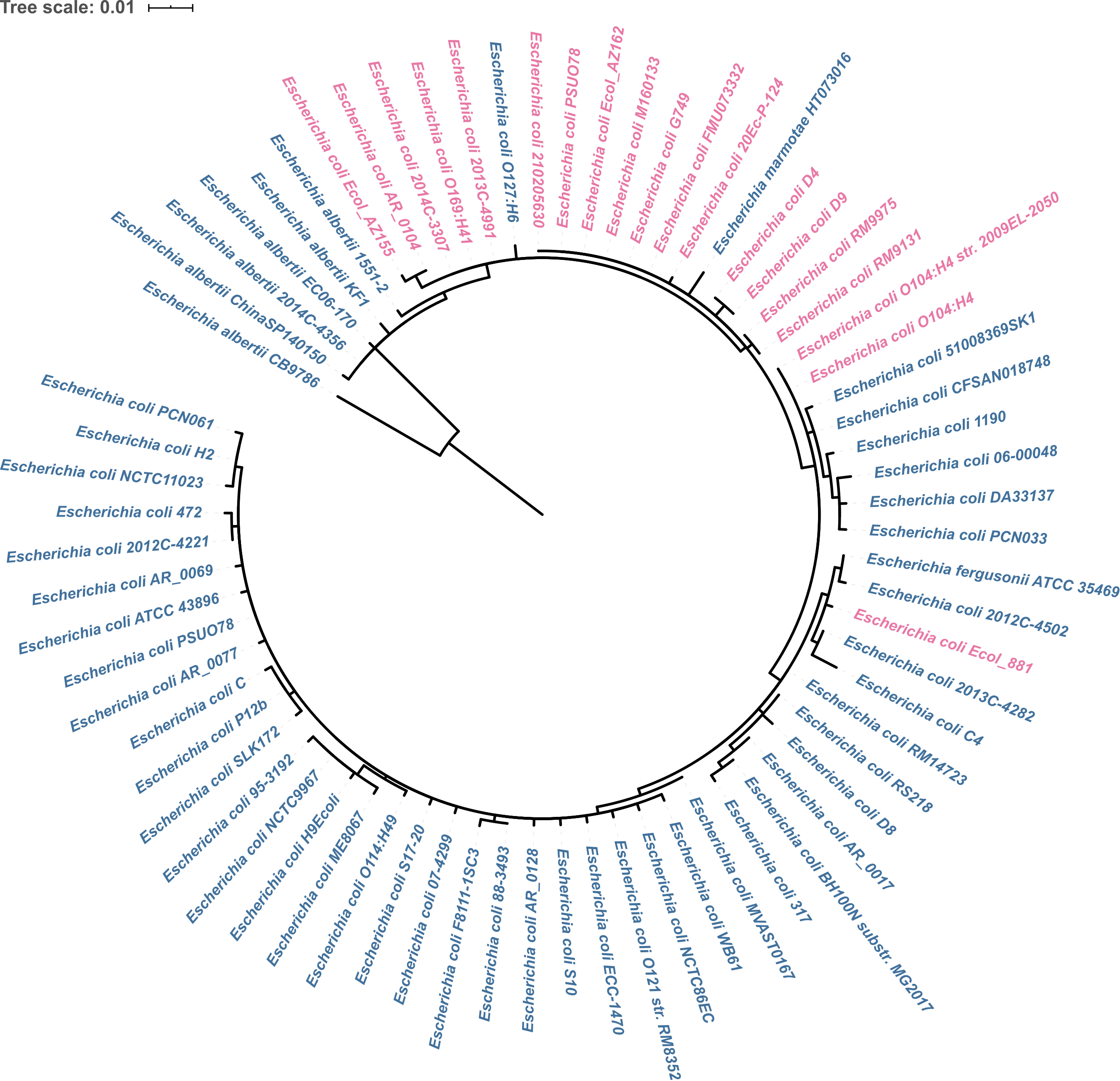

Supplement: S4 Fig — Isolate names are colored according to gene location with red for plasmids and blue for chromosomes. MetG in Escherichia coli isolate 2014C-3307 is found only on a plasmid (i.e., it has no chromosomal homolog). (TIF) [file pgen.1009656.s004.tif]

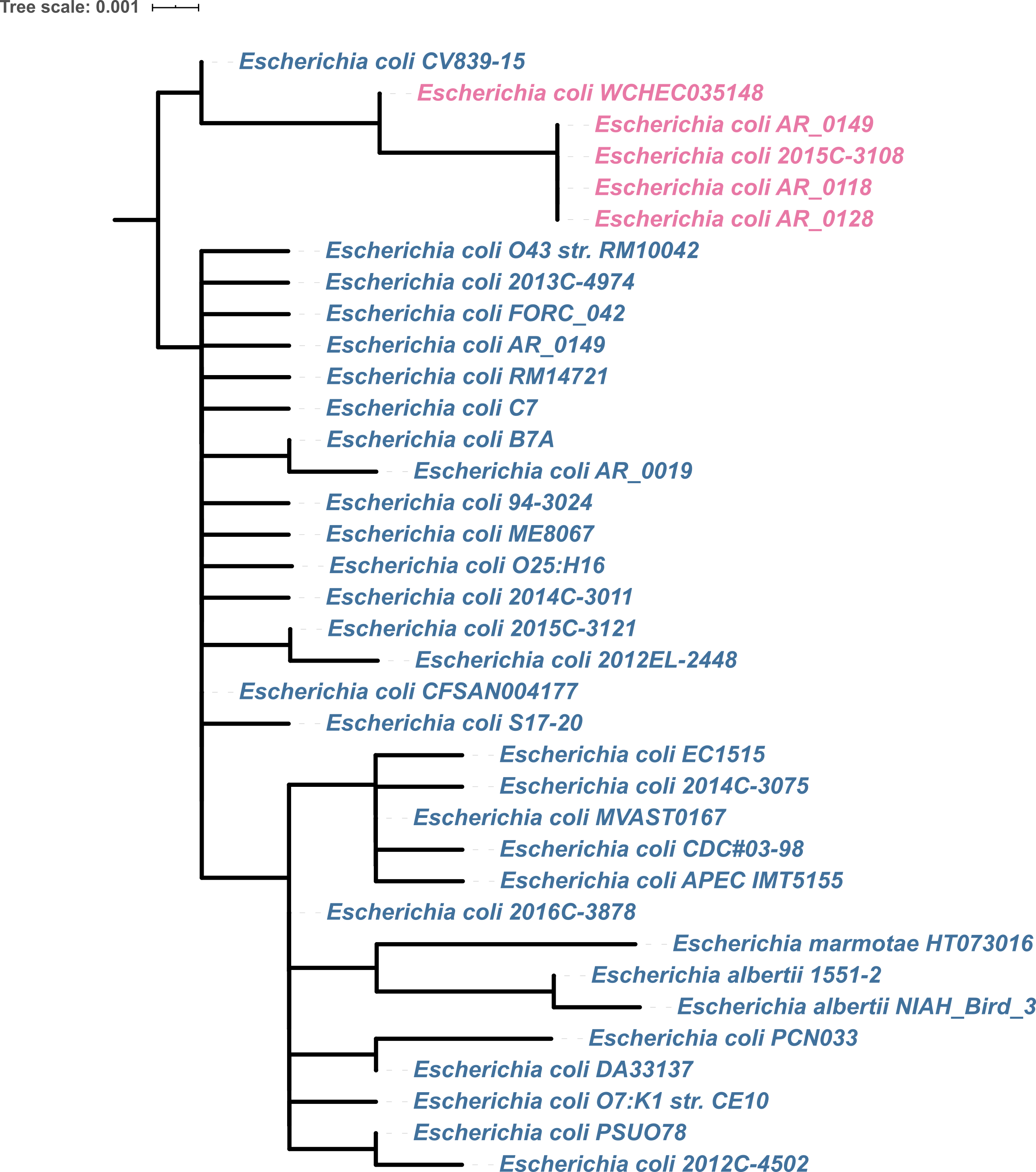

Supplement: S5 Fig — Isolate names are colored according to gene location with red for plasmids and blue for chromosomes. (TIF) [file pgen.1009656.s005.tif]

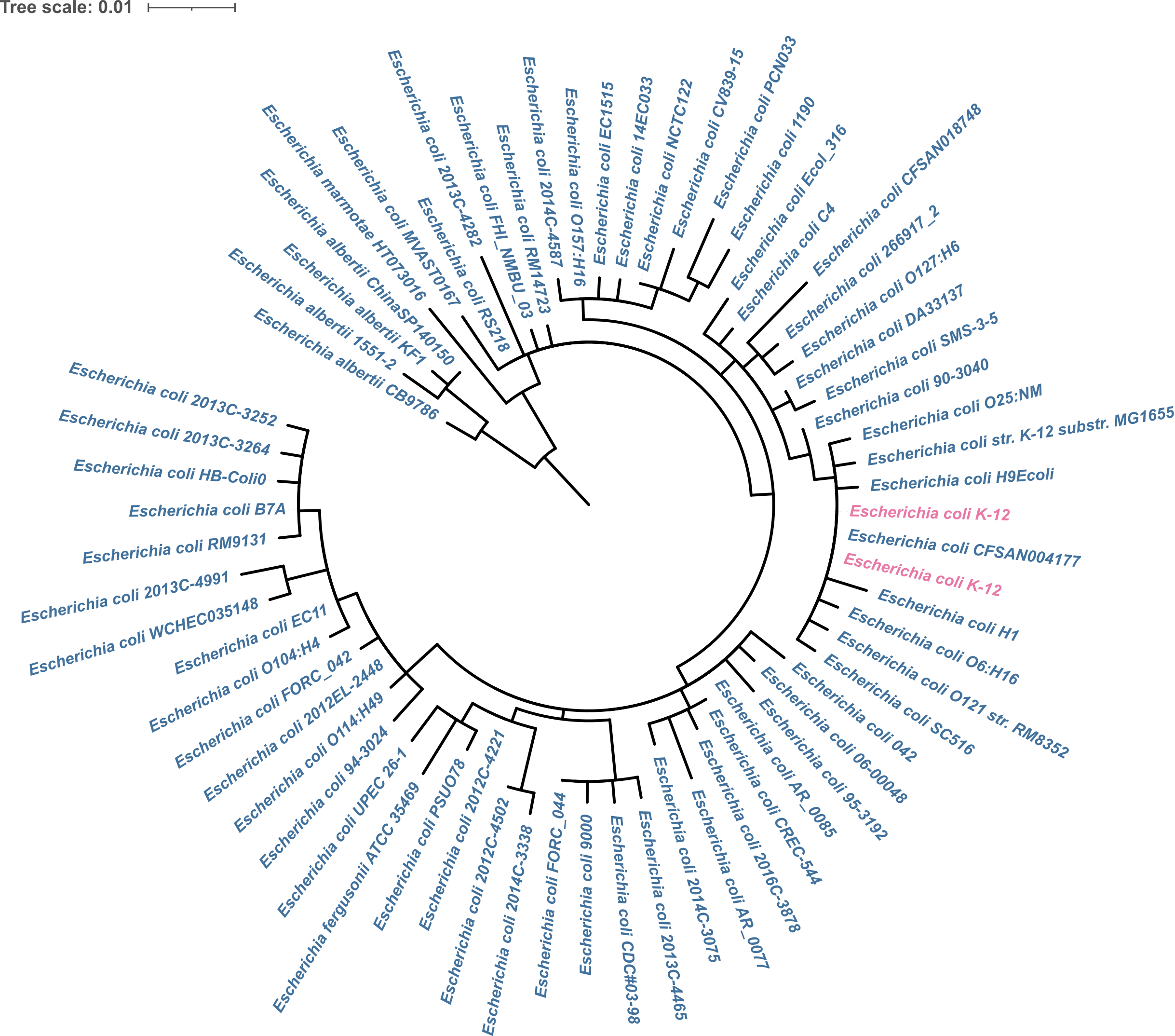

Supplement: S6 Fig — Isolate names are colored according to gene location with red for plasmids and blue for chromosomes. (TIF) [file pgen.1009656.s006.tif]

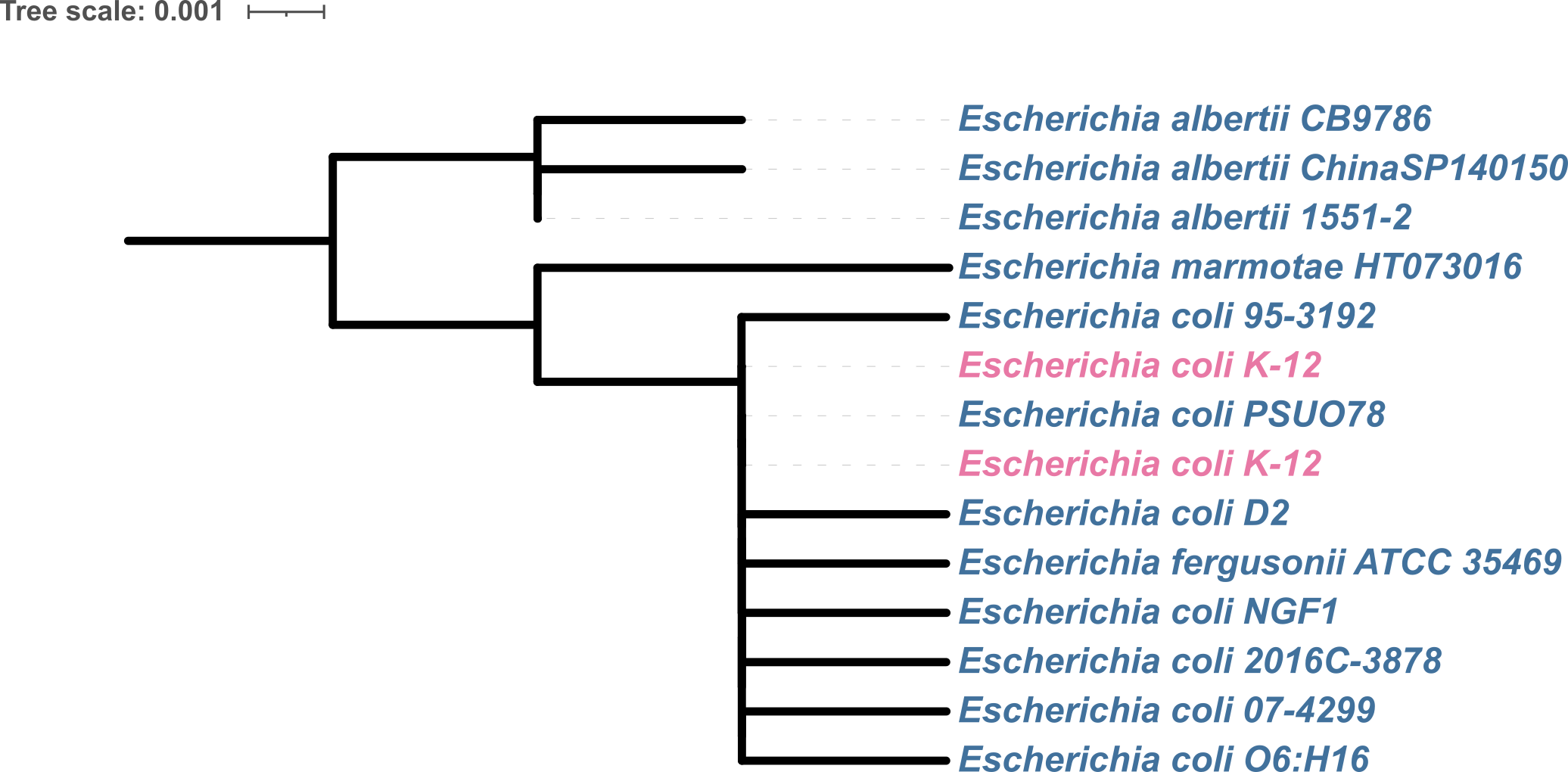

Supplement: S7 Fig — Isolate names are colored according to gene location with red for plasmids and blue for chromosomes. (TIF) [file pgen.1009656.s007.tif]

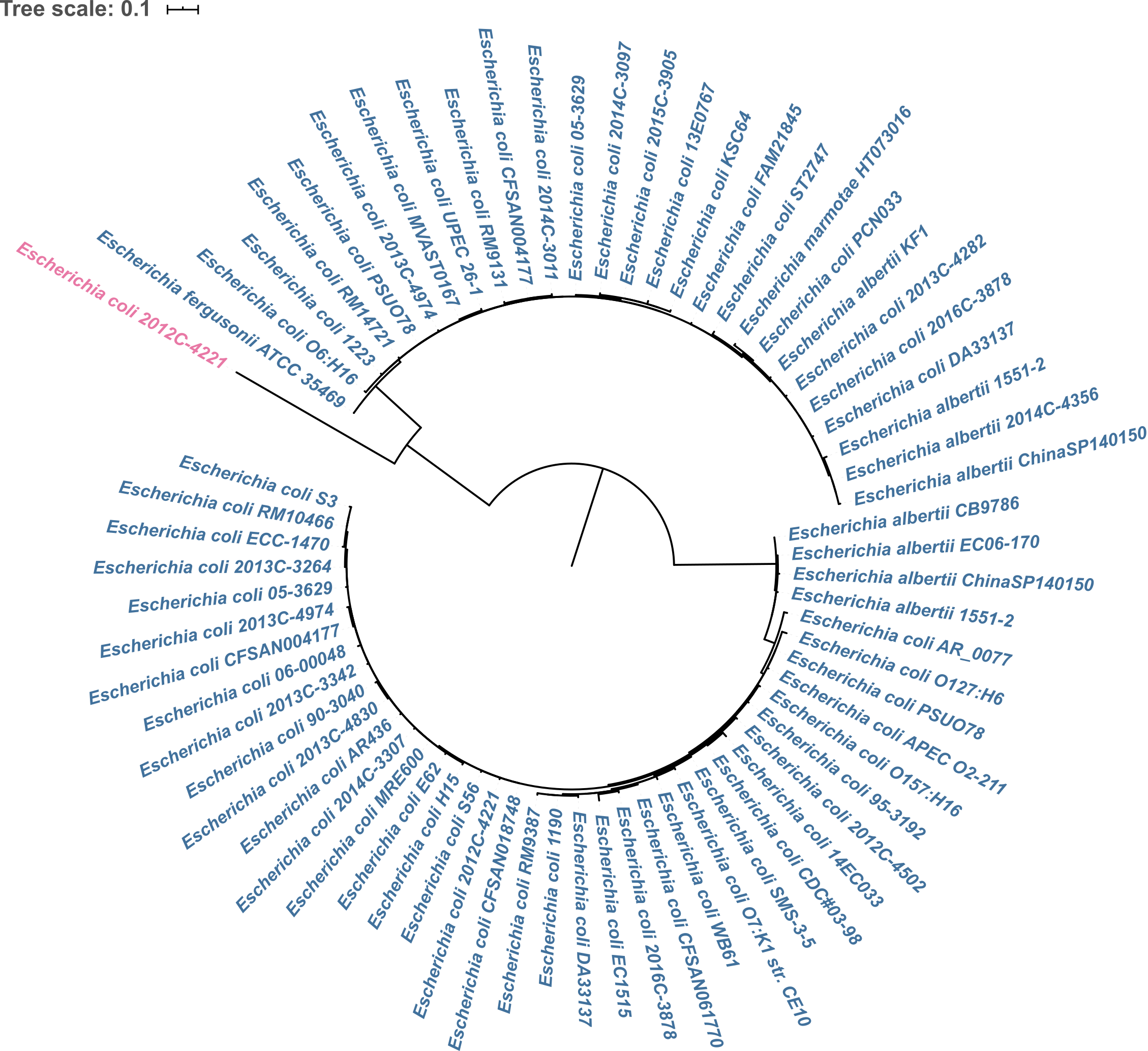

Supplement: S8 Fig — Isolate names are colored according to gene location with red for plasmids and blue for chromosomes. (TIF) [file pgen.1009656.s008.tif]

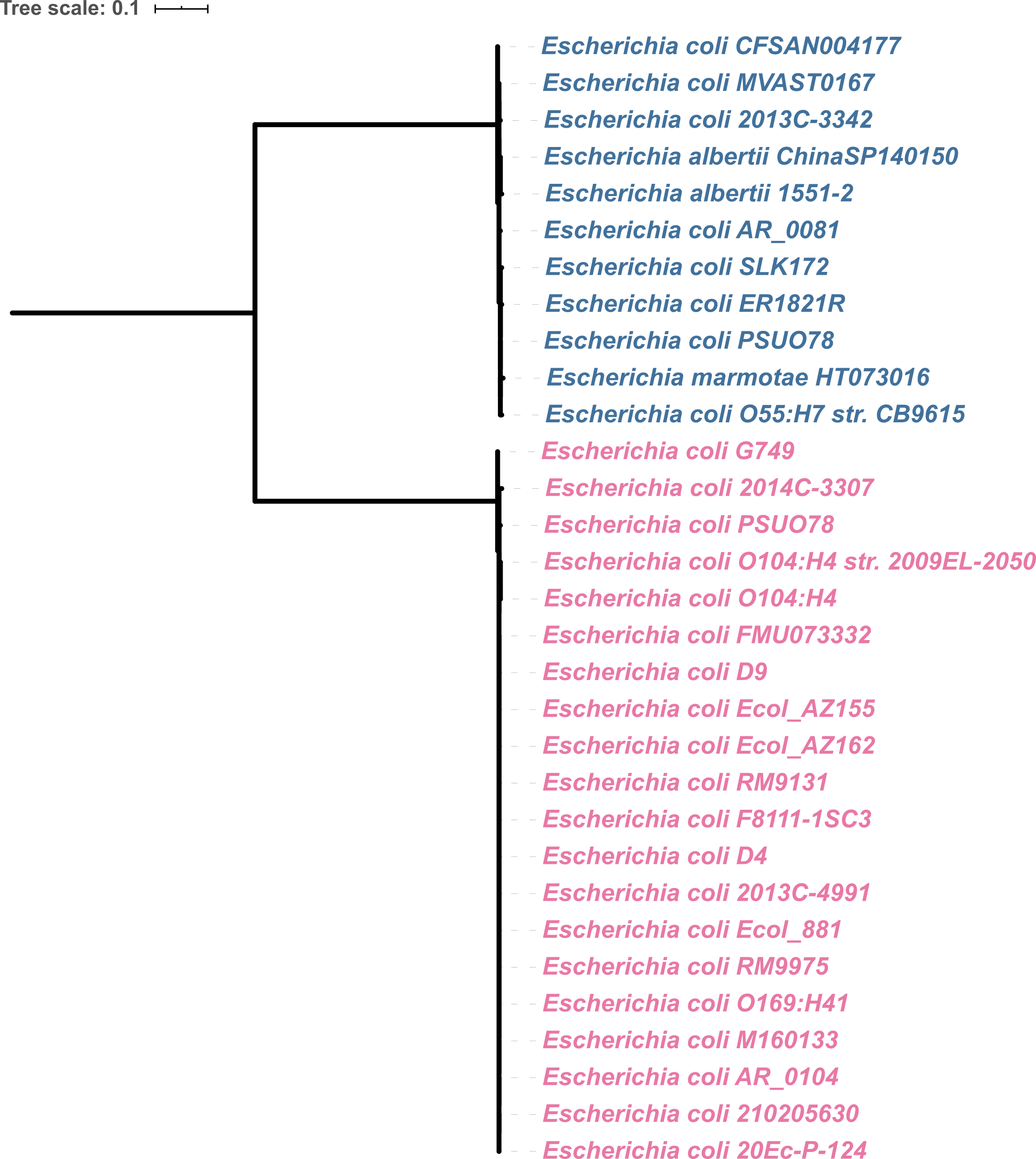

Supplement: S9 Fig — Isolate names are colored according to gene location with red for plasmids and blue for chromosomes. (TIF) [file pgen.1009656.s009.tif]

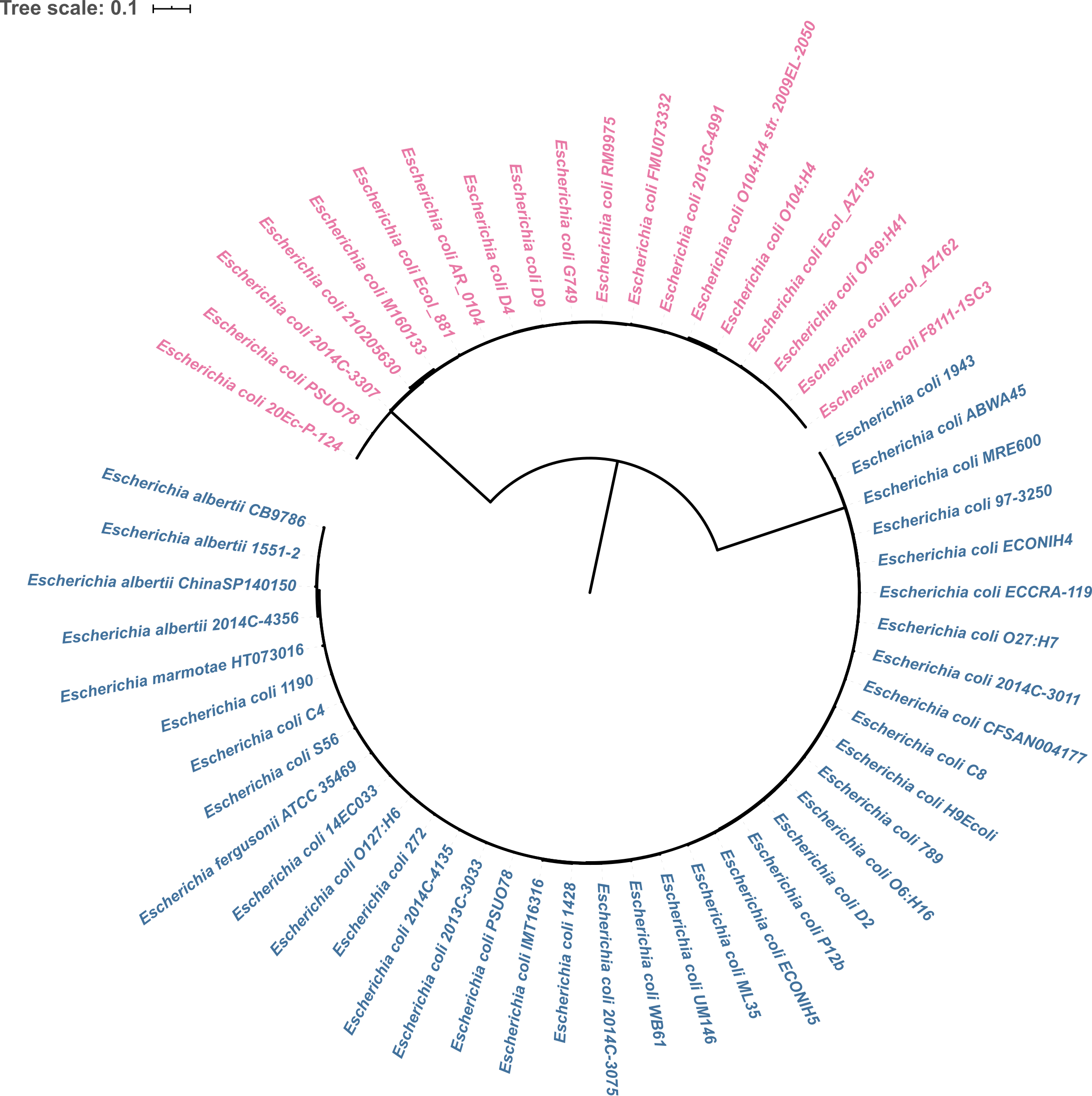

Supplement: S10 Fig — Isolate names are colored according to gene location with red for plasmids and blue for chromosomes. (TIF) [file pgen.1009656.s010.tif]

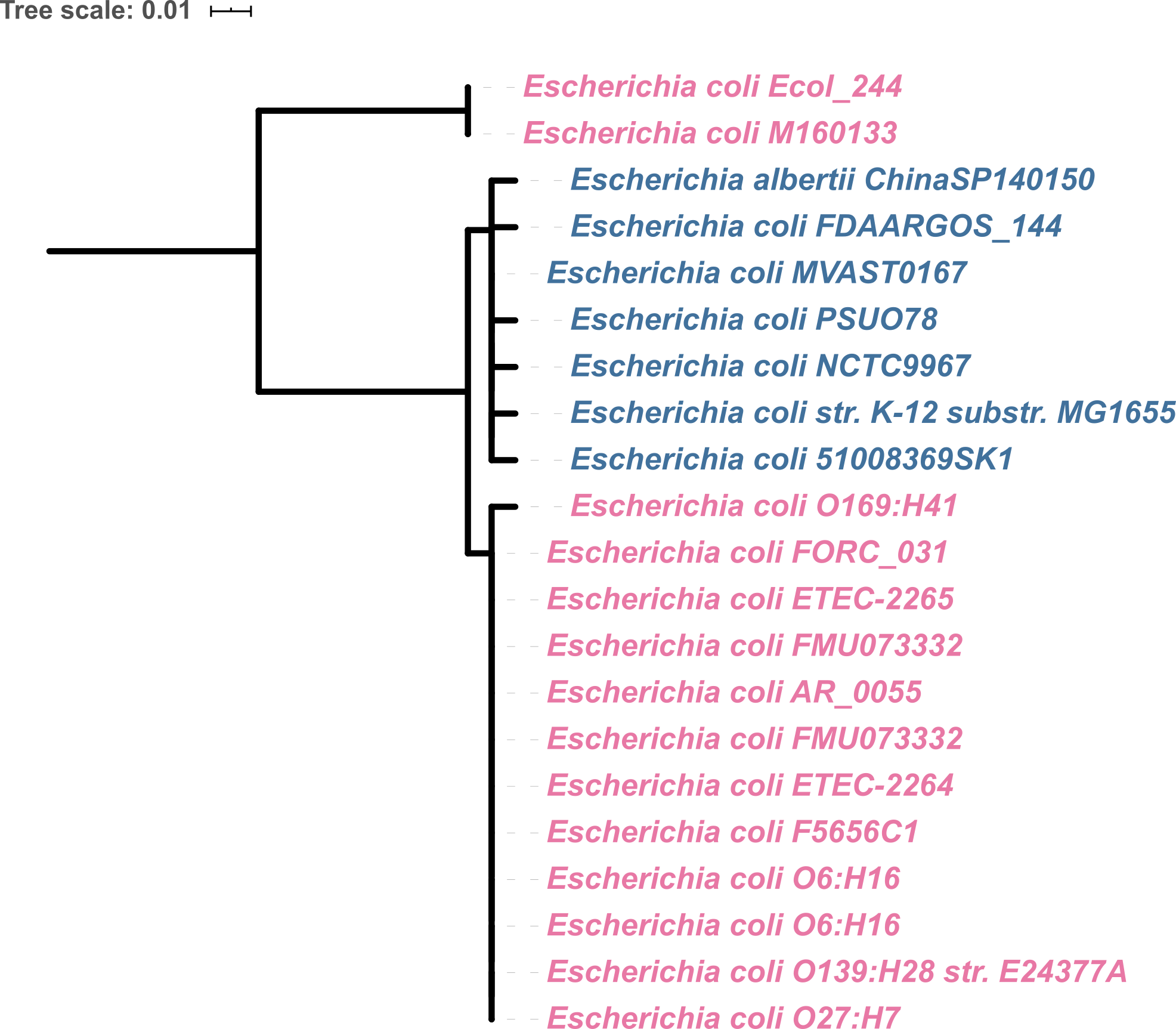

Supplement: S11 Fig — Isolate names are colored according to gene location with red for plasmids and blue for chromosomes. (TIF) [file pgen.1009656.s011.tif]

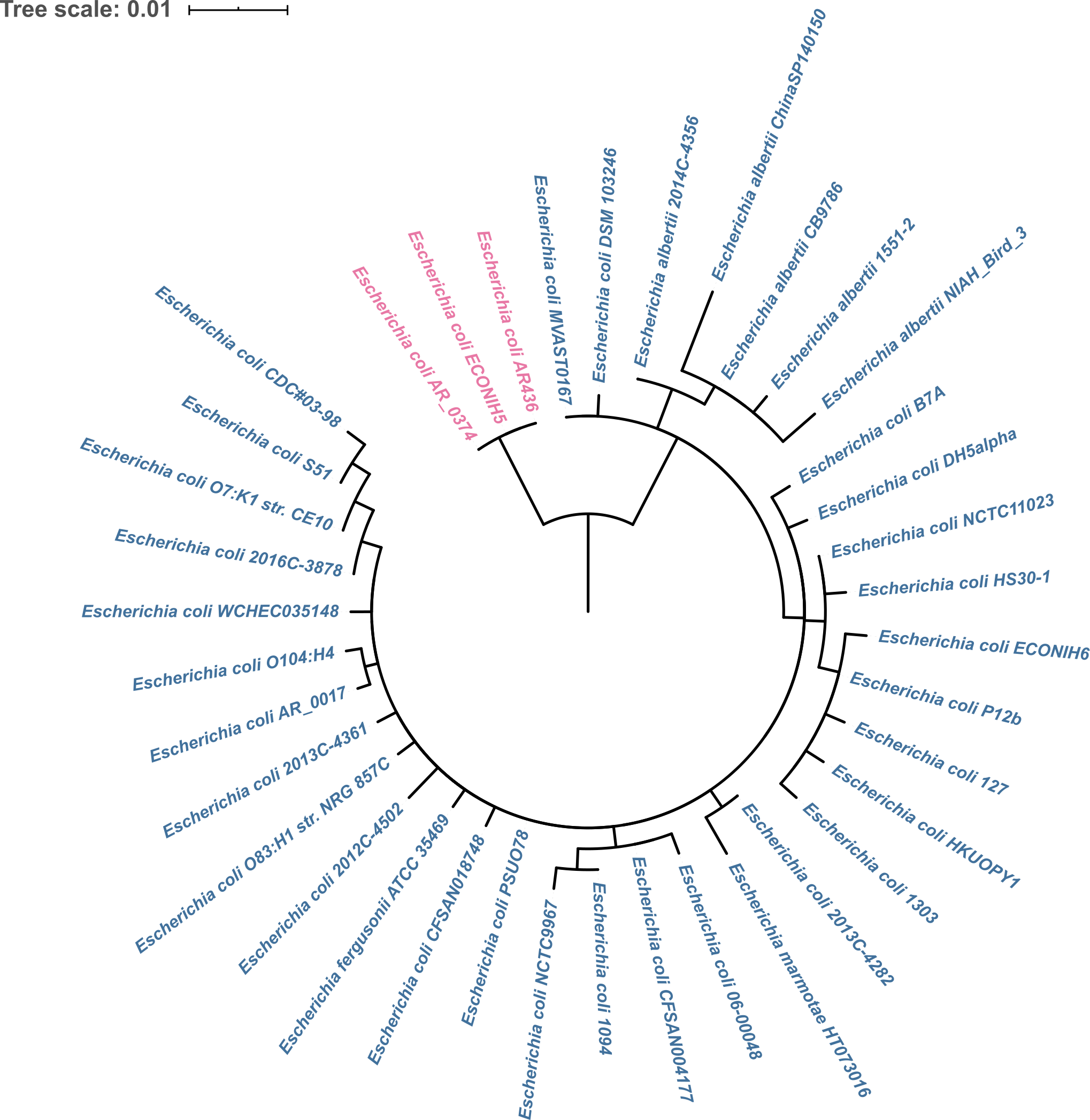

Supplement: S12 Fig — Isolate names are colored according to gene location with red for plasmids and blue for chromosomes. (TIF) [file pgen.1009656.s012.tif]

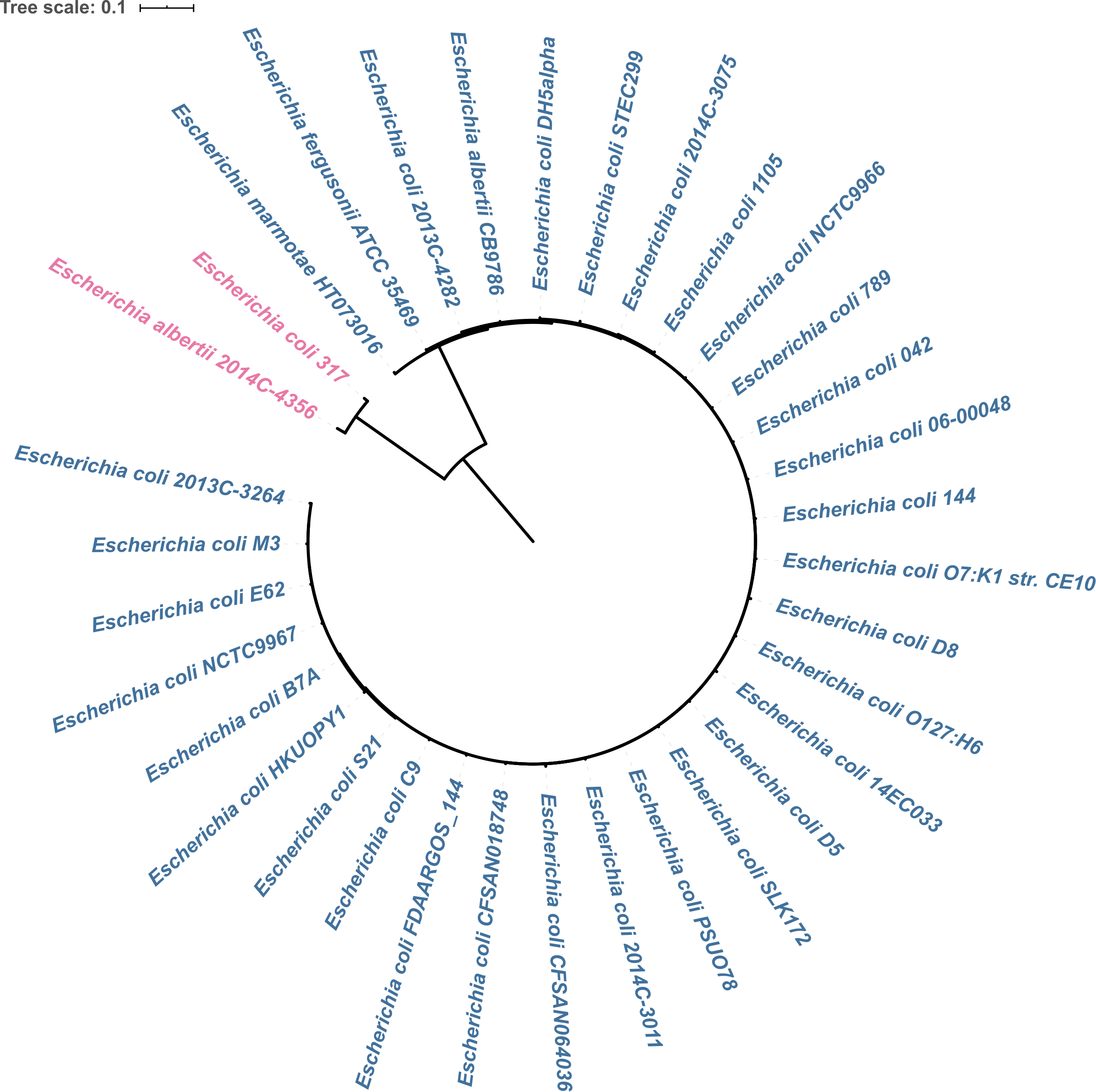

Supplement: S13 Fig — Isolate names are colored according to gene location with red for plasmids and blue for chromosomes. (TIF) [file pgen.1009656.s013.tif]

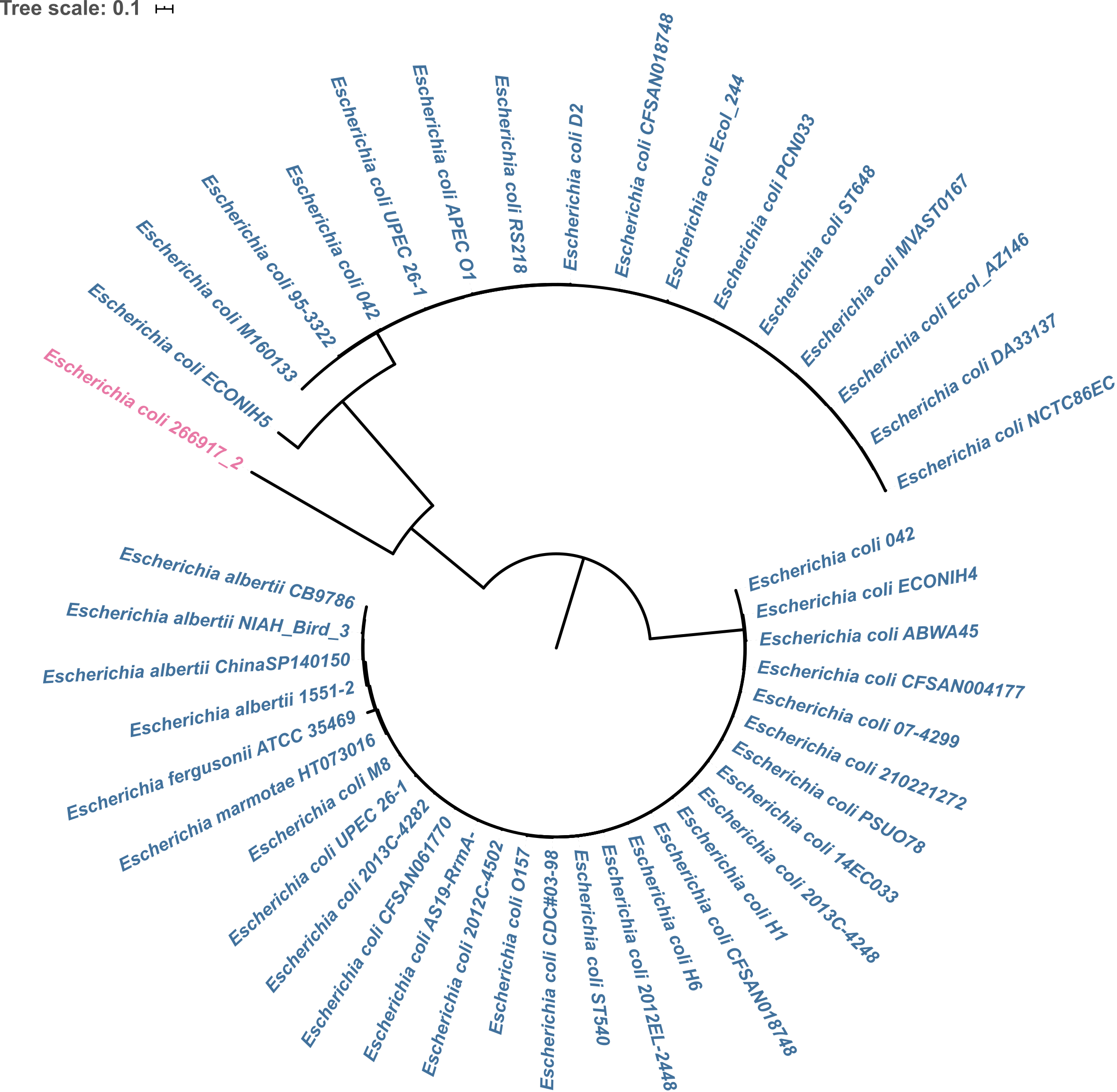

Supplement: S14 Fig — Isolate names are colored according to gene location with red for plasmids and blue for chromosomes. (TIF) [file pgen.1009656.s014.tif]

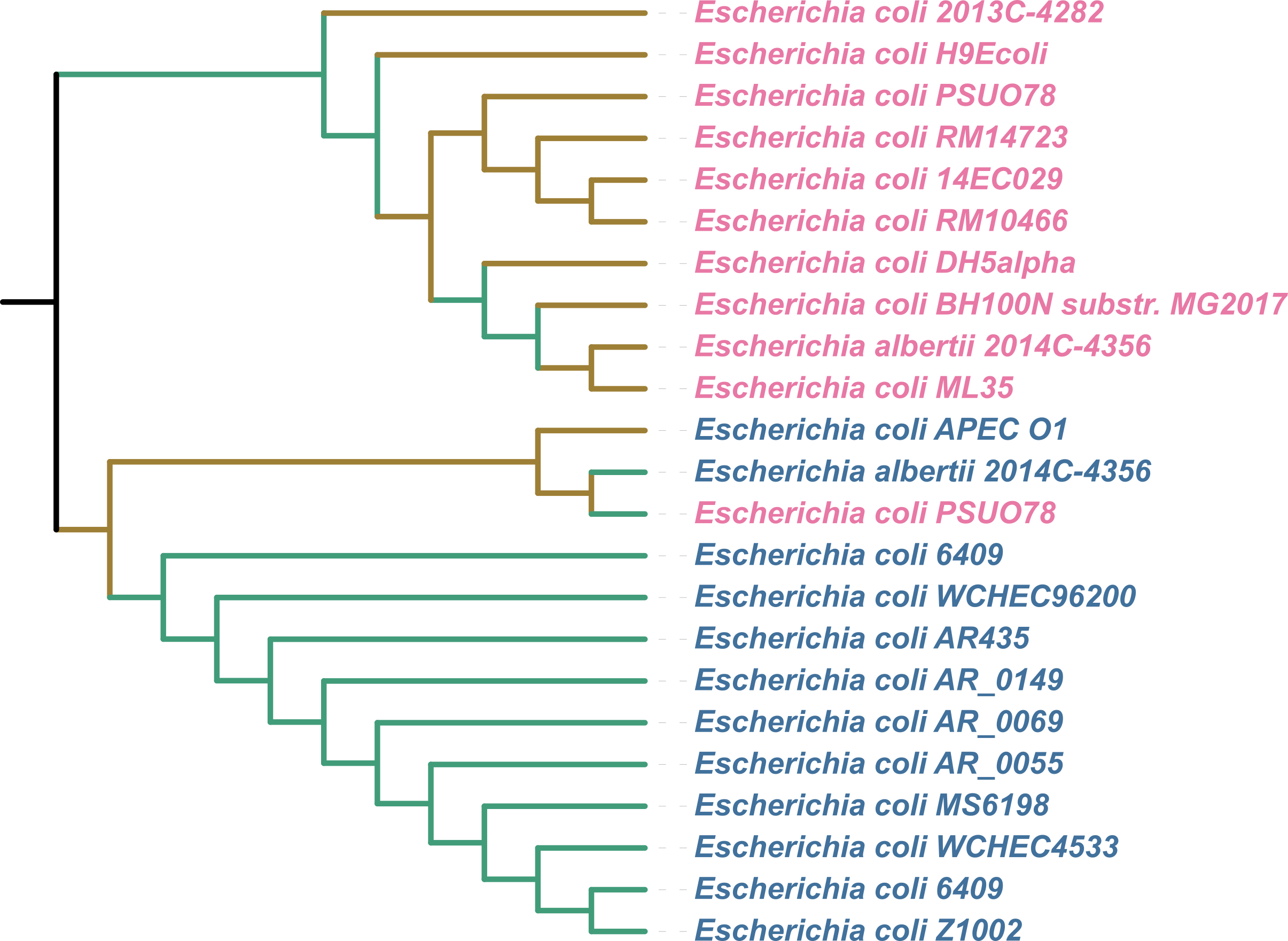

Supplement: S15 Fig — Branches are colored according to the selection intensity parameter k, ranging between brown for k<1 (relaxed selection) and green for k>1 (intensified selection). Note that branch length information is excluded in this figure. Isolate names are colored according to gene location with red for plasmids and blue for chromosomes. (TIF) [file pgen.1009656.s015.tif]

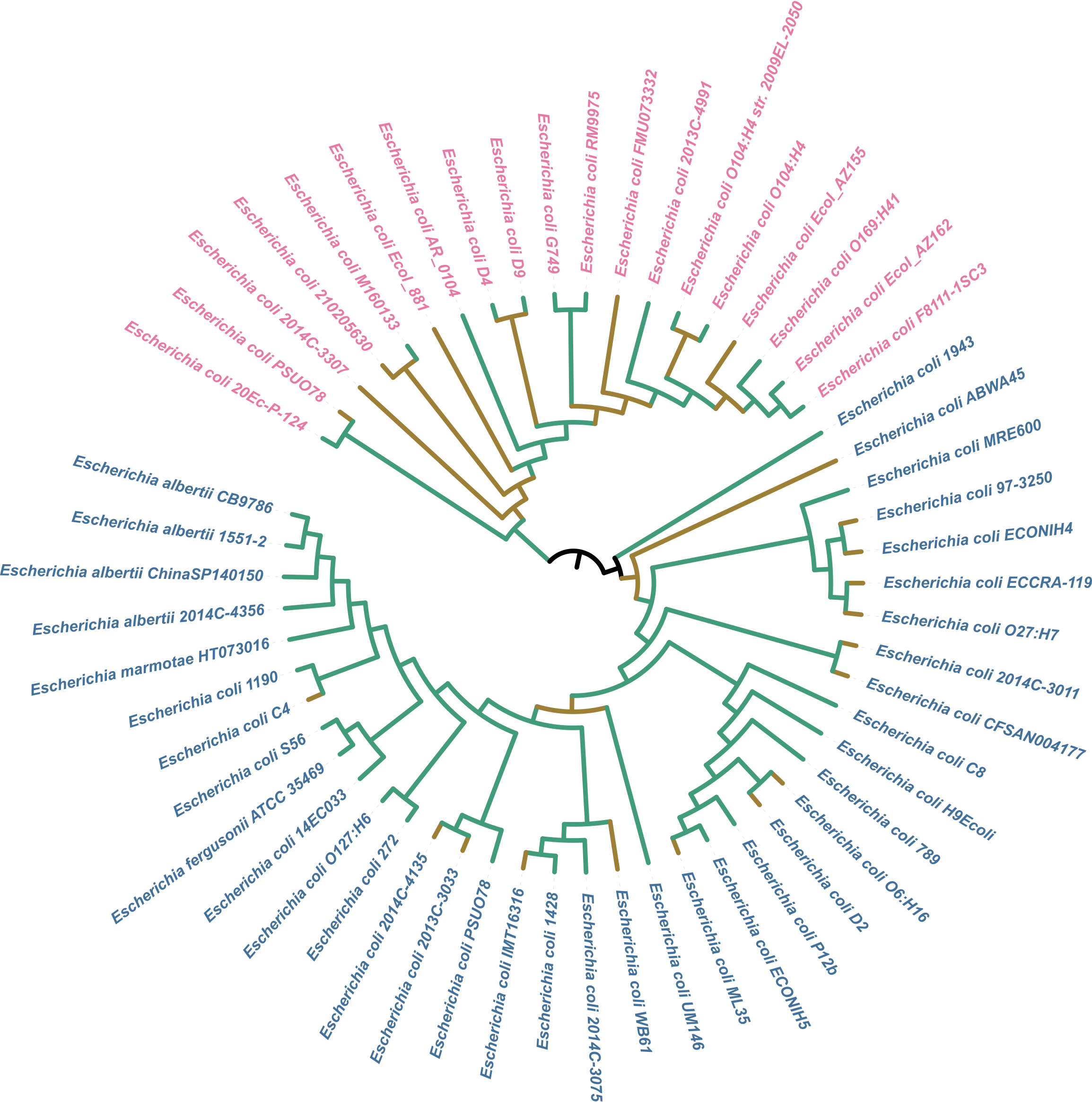

Supplement: S16 Fig — Branches are colored according to the selection intensity parameter k, ranging between brown for k<1 (relaxed selection) and green for k>1 (intensified selection). Note that branch length information is excluded in this figure. Isolate names are colored according to gene location with red for plasmids and blue for chromosomes. (TIF) [file pgen.1009656.s016.tif]

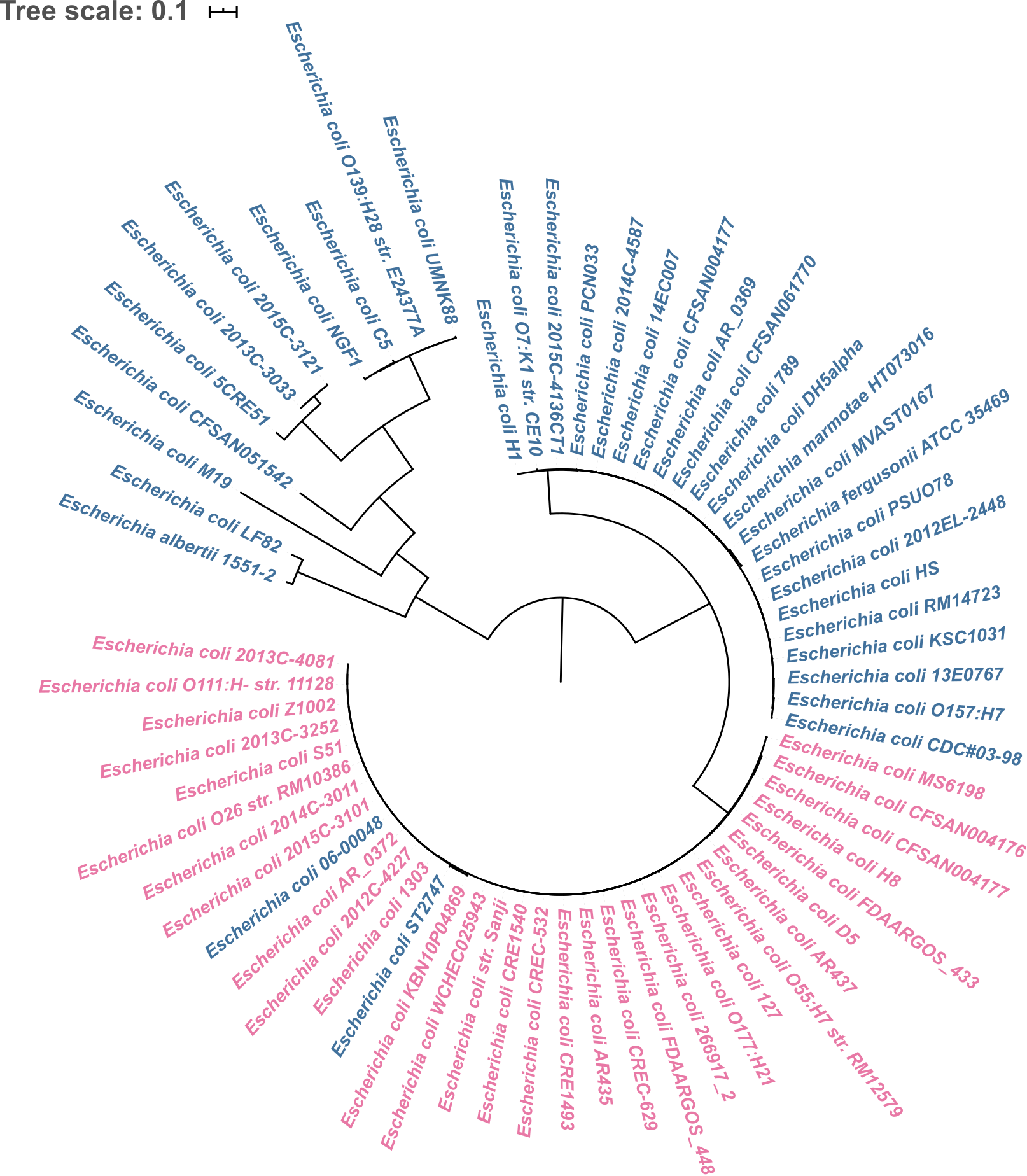

Supplement: S17 Fig — Isolate names are colored according to gene location with red for plasmids and blue for chromosomes. (TIF) [file pgen.1009656.s017.tif]

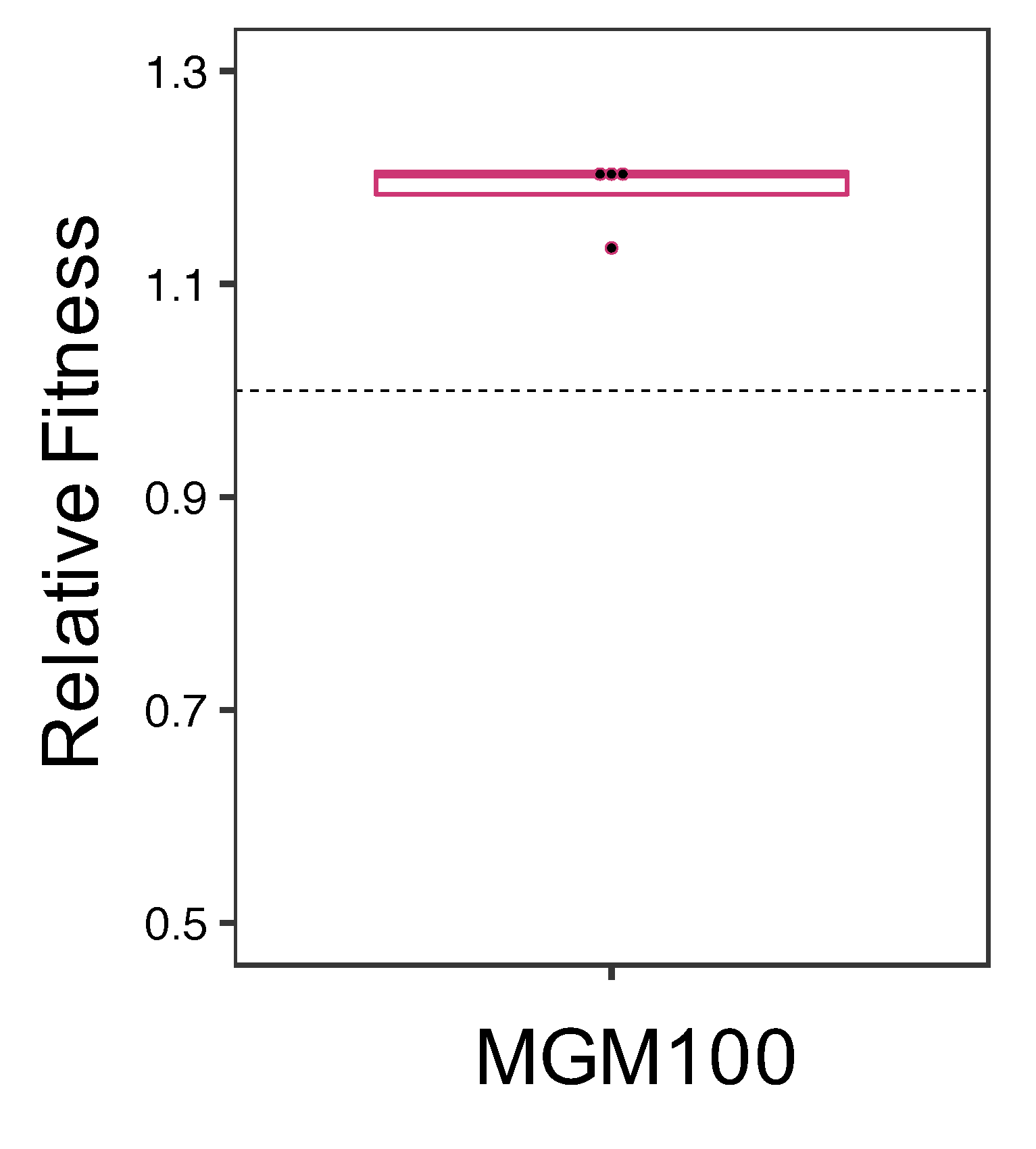

Supplement: S18 Fig — (TIFF) [file pgen.1009656.s018.tiff]

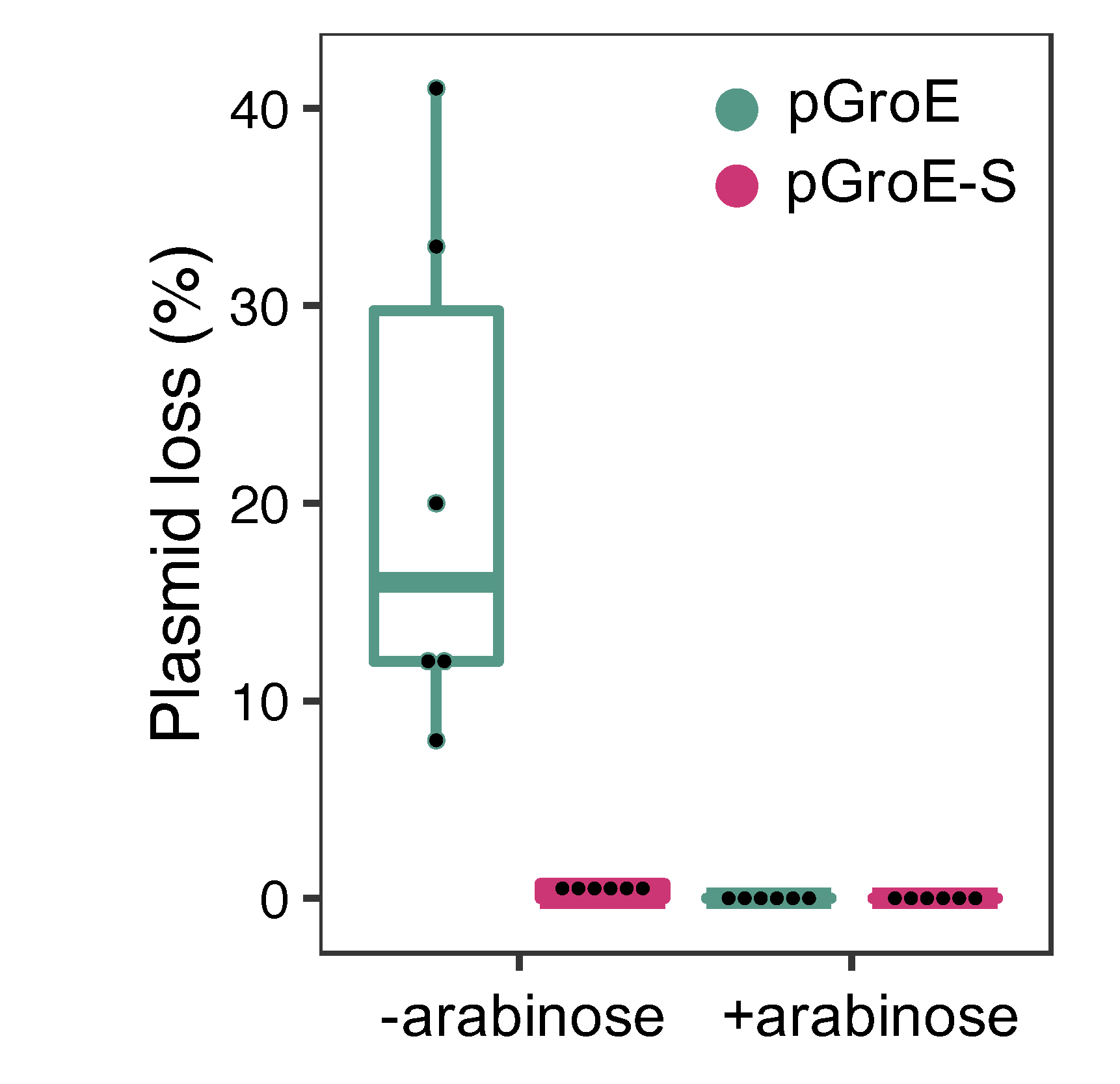

Supplement: S19 Fig — (TIFF) [file pgen.1009656.s019.tiff]

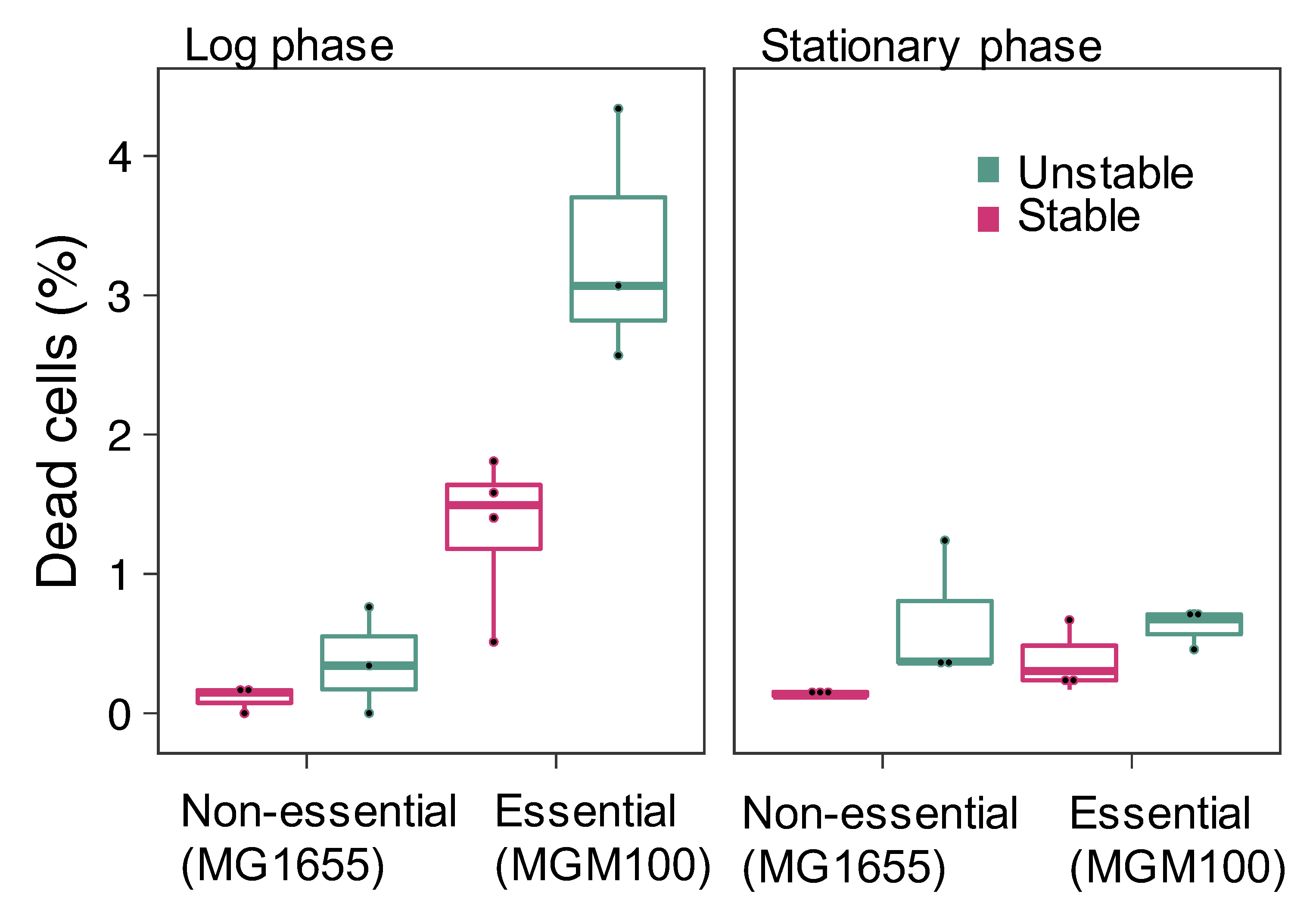

Supplement: S20 Fig — (TIFF) [file pgen.1009656.s020.tiff]

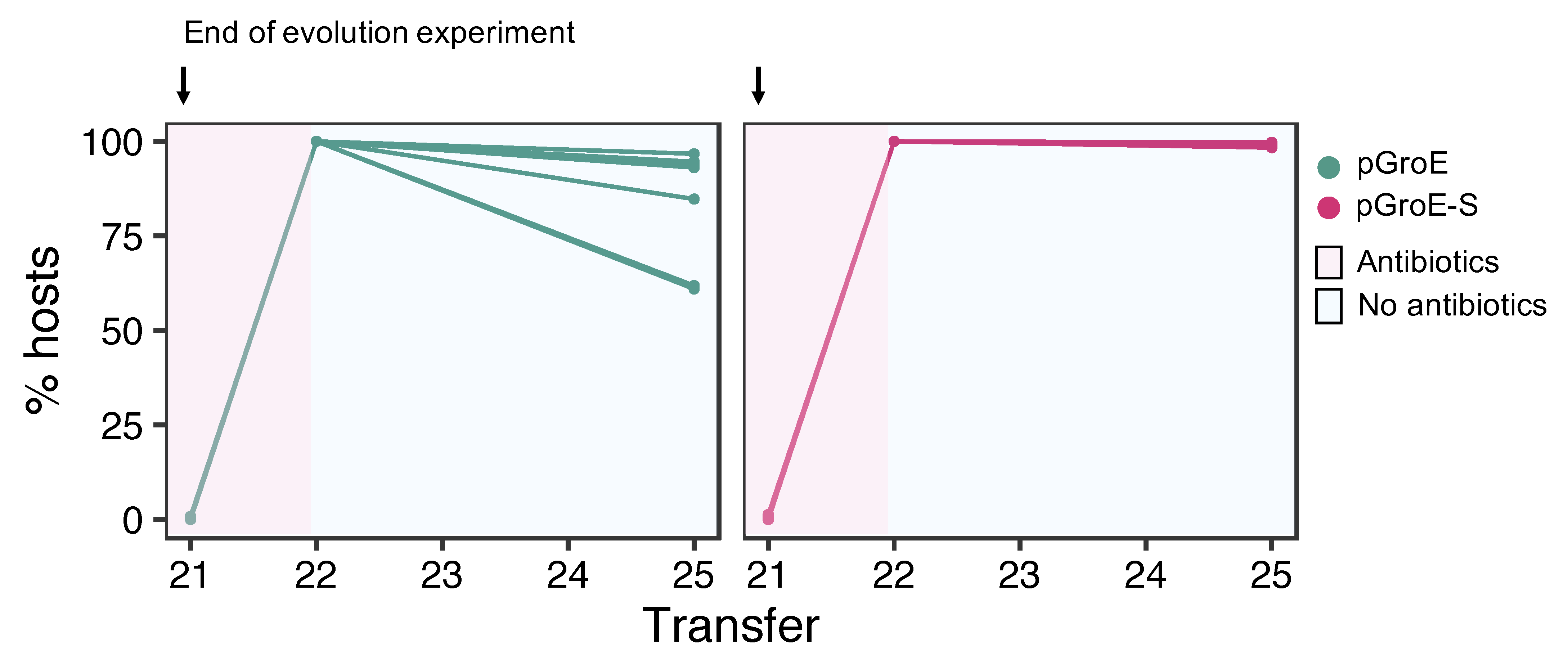

Supplement: S21 Fig — Plasmid carrying populations were exposed to a transfer in antibiotics (kanamycin 25 μg/ml). (TIFF) [file pgen.1009656.s021.tiff]

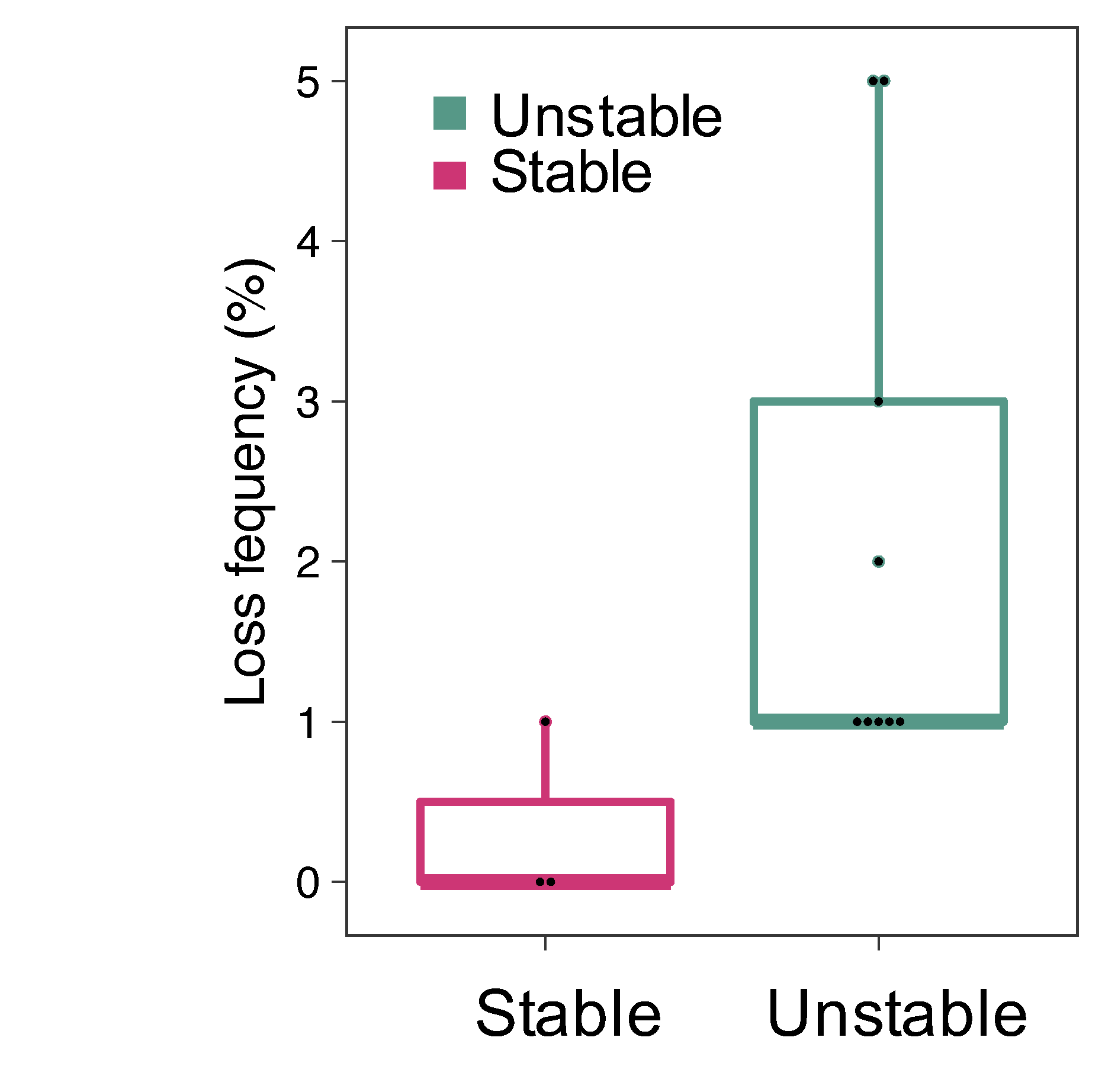

Supplement: S22 Fig — The plasmid loss was measured after overnight incubation (n = 6). (TIFF) [file pgen.1009656.s022.tiff]

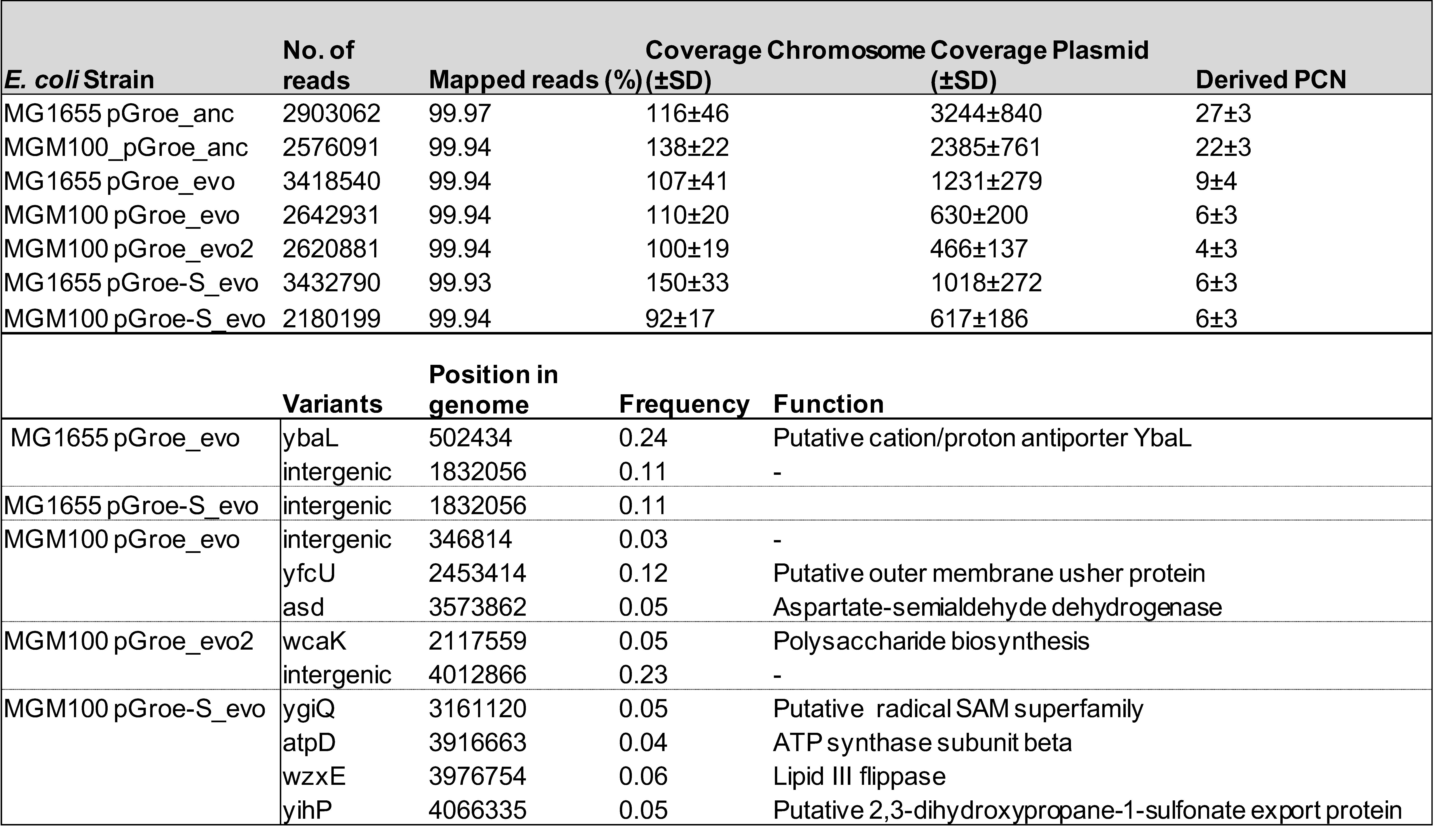

Supplement: S5 Table — Sequenced samples include ancestral populations as well as evolved strains carrying the plasmid pGroE or pGroE-S. (TIFF) [file pgen.1009656.s027.tiff]

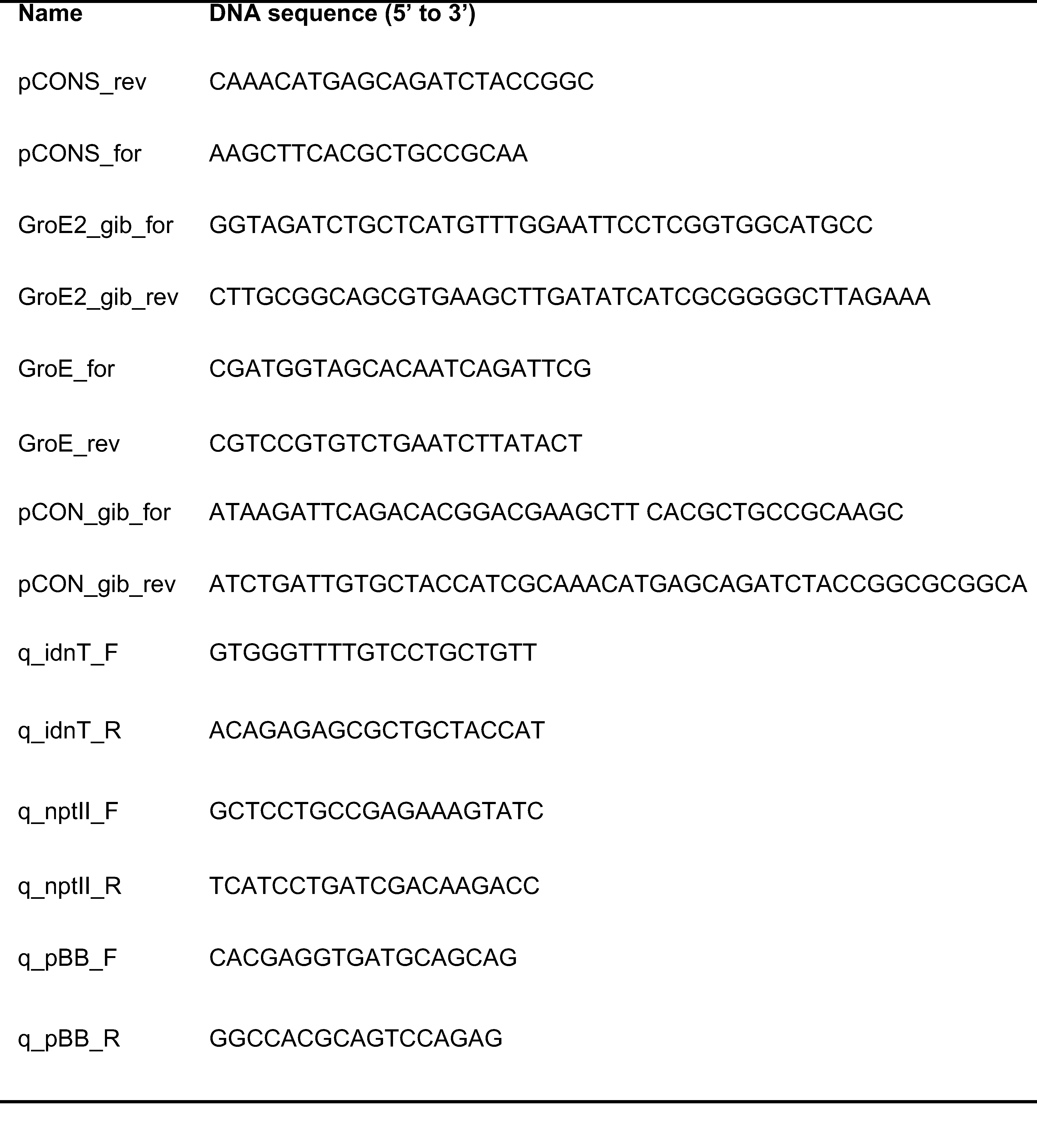

Supplement: S6 Table — (TIFF) [file pgen.1009656.s028.tiff]
